# Integrated MicroRNA-mRNA-Analysis of Human Monocyte Derived Macrophages upon *Mycobacterium avium* subsp. *hominissuis* Infection

**DOI:** 10.1371/journal.pone.0020258

**Published:** 2011-05-24

**Authors:** Jutta Sharbati, Astrid Lewin, Barbara Kutz-Lohroff, Elisabeth Kamal, Ralf Einspanier, Soroush Sharbati

**Affiliations:** 1 Institute of Veterinary Biochemistry, Freie Universitaet Berlin, Berlin, Germany; 2 Mycology/Parasitology/Intracellular Pathogens, Robert Koch-Institute, Berlin, Germany; Institute of Microbial Technology, India

## Abstract

**Background:**

Many efforts have been made to understand basal mechanisms of mycobacterial infections. Macrophages are the first line of host immune defence to encounter and eradicate mycobacteria. Pathogenic species have evolved different mechanisms to evade host response, e.g. by influencing macrophage apoptotic pathways. However, the underlying molecular regulation is not fully understood. A new layer of eukaryotic regulation of gene expression is constituted by microRNAs. Therefore, we present a comprehensive study for identification of these key regulators and their targets in the context of host macrophage response to mycobacterial infections.

**Methodology/Principal Findings:**

We performed microRNA as well as mRNA expression analysis of human monocyte derived macrophages infected with several *Mycobacterium avium hominissuis* strains by means of microarrays as well as quantitative reverse transcription PCR (qRT-PCR). The data revealed the ability of all strains to inhibit apoptosis by transcriptional regulation of BCL2 family members. Accordingly, at 48 h after infection macrophages infected with all *M. avium* strains showed significantly decreased caspase 3 and 7 activities compared to the controls. Expression of let-7e, miR-29a and miR-886-5p were increased in response to mycobacterial infection at 48 h. The integrated analysis of microRNA and mRNA expression as well as target prediction pointed out regulative networks identifying caspase 3 and 7 as potential targets of let-7e and miR-29a, respectively. Consecutive reporter assays verified the regulation of caspase 3 and 7 by these microRNAs.

**Conclusions/Significance:**

We show for the first time that mycobacterial infection of human macrophages causes a specific microRNA response. We furthermore outlined a regulatory network of potential interactions between microRNAs and mRNAs. This study provides a theoretical concept for unveiling how distinct mycobacteria could manipulate host cell response. In addition, functional relevance was confirmed by uncovering the control of major caspases 3 and 7 by let-7e and miR-29a, respectively.

## Introduction

Mononuclear phagocytes represent gateways of the host immune system for encountering and eliminating pathogens. However, various disease agents have evolved efficient strategies to overcome this fundamental mechanism of the innate immunity. For example, the genus *Mycobacterium* comprises highly pathogenic species but also opportunists such as *M. avium*, which are able to withstand the hostile phagosomal environment and cause disseminated infections. Mycobacterial infections of humans and animals give cause for serious concern. In this regard, tuberculosis remains one of the major threats to humans still possessing a high mortality rate. But also less pathogenic mycobacteria e.g. members of the *M. avium* complex (MAC) are able to cause disseminated infections in immuno-compromised persons such as AIDS patients [1]. MAC furthermore elicits lymphadenopathies in otherwise healthy children and pneumonia in persons with pre-disposing lung conditions [2], [3]. MAC comprises the species *M. intracellulare* and *M. avium*. The latter is divided into four subspecies: *M. avium* subsp. *hominissuis* (MAH), *M. avium* subsp. *avium* (MAA), *M. avium* subsp. *silvaticum* (MAS) and *M. avium* subsp. *paratuberculosis* (MAP). Human mycobacteriosis is predominantly caused by MAH, while MAA and MAS affect primarily birds [4] and MAP is the causative agent of Johne's disease.

*M. avium* is phagocytosed by macrophages after binding to the CR3 complement receptor, the vitronectin receptor, the mannose receptor, CD14, CD43 and Toll-like receptors (TLR) [5], [6]. Recognition of *M. avium* by TLR2 and TLR4 initiates a MyD88-dependent activation of antibacterial effector mechanisms [6], [7]. In monocyte derived macrophages (MDMs) both TLR2 and TLR4 induce e.g. tyrosine phosphorylation of phospholipase C gamma 2 (PLCG2) causing the release of Ca^2+^ and production of pro-inflammatory cytokines such as tumor necrosis factor (TNF) [8]. Nonetheless, *M. avium* is able to inhibit the phagosome-lysosome-fusion and to replicate within macrophages. Survival of *M. avium* within phago-lysosomes of macrophages may be explained by reduced response to interferon gamma (IFNG) upon infection. This may result from up-regulation of the suppressors of cytokine signalling (SOCS) upon interaction of macrophage receptors with *M. avium*. Increased SOCS1 and SOCS3 expression was demonstrated as early as two hours after infection of macrophages with *M. avium* [9]. Virulent strains of *M. avium* activate macrophages to a lower degree compared to non-virulent strains [10]. Interestingly, pronounced interleukin (IL) 12 expression was shown after infection with non-virulent strains. It was suggested that virulence could be considered as the intrinsic inability of certain isolates to activate macrophages. Moreover, it was shown that infection of human MDMs with *M. avium* causes an early response (after 2 h) of signalling molecules and transcription factors including the mitogen-activated protein kinase (MAPK) pathway. Also NFκB mediated pro-inflammatory response is triggered early after *M. avium* infection together with up-regulation of several matrix metalloproteinases (MMPs), which also possess increased expression after 24 h infection. The NFκB activation together with increased expression of TNF and IL1B drive a pro-inflammatory response to combat *M. avium*, while early but non-sustained IL10 and transforming growth factor beta (TGFB) expression seem to weaken this effect [6]. TNF and IL1B exert pro-inflammatory effects such as recruitment of neutrophils, activation of monocytes or induction of endothelial adhesion molecules. It was however shown that IL10 production in macrophages infected with *M. avium* results in inhibition of TNF mediated apoptosis [11]. Apoptosis appears after TNF mediated activation of the extrinsic pathway leading to caspase 8 (CASP8) activation. Concomitant permeabilisation of the mitochondrial membrane activates the intrinsic apoptotic pathway. Both pathways result in CASP3 activation and induction of apoptosis [12], [13]. Programmed cell death of infected host cells is an archaic mechanism of the immune system to eradicate pathogens. Behar and colleagues (2010) reviewed that attenuated *M. tuberculosis* induces macrophage apoptosis while virulent strains inhibit apoptosis and induce necrosis. The latter is a characteristic being absent in non-pathogenic mycobacteria. Apoptosis of infected macrophages is associated with diminished pathogen viability and has the benefit for the host to constrain the infection, to minimise tissue injury and can promote cross-priming. On the other hand, inhibition of cell death allows the pathogen to evade humoral immunity and provides time for the pathogen to replicate [12].

A number of studies have reported differential gene expression of macrophages upon infection with *M. avium* [6], [10], [14]. In general, regulated genes were involved in production of cytokines, lymphokines, chemokines, in adhesion, signal transduction, transcription, protein cleavage and actin polymerisation. Recent studies have indicated that many of the mentioned signalling molecules are under the control of microRNAs (miRNAs). MiRNAs belong to the class of regulating small non-coding RNAs directing mRNA degradation following deadenylation or translational repression. Current research on miRNAs has unveiled a new layer of eukaryotic gene expression regulation unravelling several unsolved biological mechanisms in development or disease. MiRNAs act as major regulators of developmental timing, cellular differentiation, signalling pathways but also apoptosis [15]. Both the extrinsic as well as the intrinsic apoptotic pathways are regulated by miRNAs. In this context, the pro-survival factor BCL2 belonging to the intrinsic pathway was reported to be targeted by miR-15 and miR-16, while miR-221 and miR-222 affect the extrinsic apoptotic pathway [16]. But also let-7a was shown to inhibit apoptosis by targeting the executioner of programmed cell death CASP3 [17]. Studies have shown that innate as well as adaptive immune cells possess specific miRNA expression patterns regulating both cell fate and function [18]. MiRNAs not only regulate early haematopoietic cell development but also direct the differentiation of common myeloid progenitor cells into different myeloid cell types. For example, monocyte, macrophage and dendritic cell (DC) development is regulated by miRNAs. Granulocyte-monocyte progenitors derive from common myeloid progenitors to produce neutrophils and monocytes. The latter can differentiate to macrophages or DCs. This process is initiated by decreased expression of the members of the miR-17-92 cluster leading to the depression of the transcription factor RUNX1 promoting monocyte differentiation. On the other hand, macrophages encountering pathogens respond with up-regulation of e.g. miR-155 or and miR-146. This response is triggered by TLR signalling as well as pro-inflammatory cytokines e.g. TNF by activating the transcription factors AP1 and NFκB. Pro-inflammatory cytokines are key molecules of pathogen eradication, however their excessive production leads to tissue damage. The role of miRNAs in the macrophage inflammatory response seems to be the boost of a sharp and intense reaction towards eradication of pathogens while avoiding excessive damage by realising a negative feedback loop [18]. During the macrophage inflammatory response miR-155 targets for example SOCS1 and SHIP1, both negative regulators of TLR signalling. On the other hand convergent expression of miR-146 affects downstream TLR signalling molecules such as interleukin-1 receptor-associated kinase (IRAK) 1 and 2 or TNF receptor-associated factor (TRAF) 6, which promote inflammation [18].

Many efforts have recently been made to understand the role of miRNAs in innate immunity and in macrophage inflammatory response to pathogens. The particular nature of the macrophage response to *Mycobacterium* infections motivated us to perform an integrated analysis of miRNAs and mRNAs upon infection of human MDMs. Based on the growing number of studies showing that miRNAs rather direct mRNA degradation than translational repression, bioinformatic tools e.g. MAGIA [19] or MMIA [20] were developed to facilitate the detection of functional miRNA-mRNA-interactions in different contexts. For our analysis, we used two MAH strains (originally isolated from human patients) to perform microarray experiments. We extended our investigations by qRT-PCR analysis and by using an additional MAH strain isolated from water. These strains were used for infection experiments with macrophages derived from buffy coats of three independent donors. Taken together, our data show for the first time that miRNAs respond specifically to mycobacterial infections compared to the control *E. coli* K12. We furthermore provide an analytical concept for dissecting the host response upon pathogen interaction. We show that inhibition of apoptosis after mycobacterial infection is directed via CASP3 and 7 down-regulation mediated by let-7e and miR-29a, respectively.

## Results

For performing the microarray based integrated analysis of mRNA and miRNA data, we chose two MAH strains being originally isolated from human patients, each as an representative for the causative agent of disseminated infections in AIDS patients or lymphadenitis, respectively. MAH strain 104 was isolated from an AIDS patient [21] and its genome is entirely sequenced. MAH strain 10091/06 was isolated from the lymph node of an infected child. MDMs used for infection experiments were enriched from buffy coats and the percentage of MDMs after enrichment amounted to around 70% as evaluated after detection of the CD14^+^ population by means of FACS analysis. Each infection was performed independently with MDMs from three different donors. For both, detection of mRNAs as well as miRNAs by means of microarray analysis, pools of total RNA from these three experiments were prepared for each MAH strain and non-infected controls at 6, 24 and 48 h post infection (p.i.). To verify linear amplification of pooled RNA used for cDNA microarrays, we determined the Pearson correlation coefficient (Pearson r) of five randomly selected genes (ACTB, BCL2, GAPDH, TNF and TP53) by performing qRT-PCR reactions before and after RNA amplification. For all selected genes we obtained clear linearity with Pearson r ranging between 0.87 (ACTB) and 0.99 (TNF) with two-tailed P values≤0.002.

### Microarray study of infected MDMs reflects a comparable response to MAH as to other virulent mycobacteria

**Figure 1:**
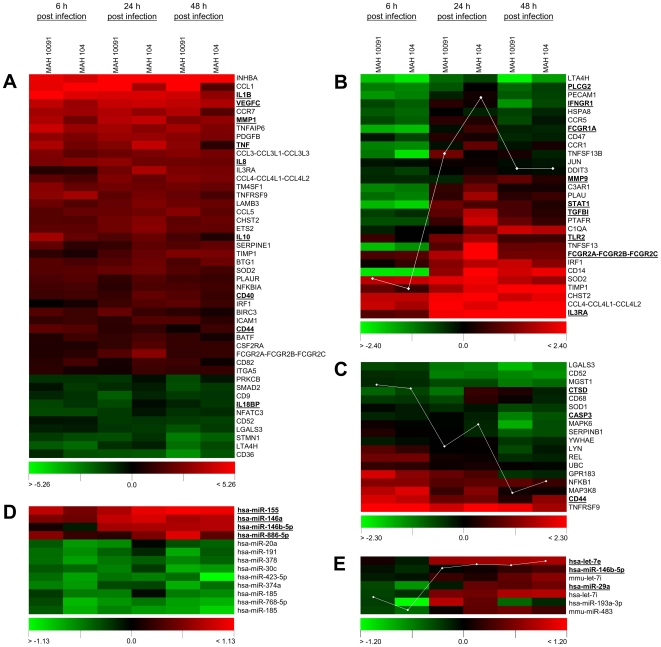
Heatmaps of mRNA expression data of MAH infected MDMs after microarray analysis. Columns represent temporal expression of MDMs at 6, 24 and 48 h p.i. with MAH strains 10091/06 and 104. Colours represent log 2 ratios of the infected cells versus the non-infected control according to the scales shown below. Samples represent a pool of three independent infection experiments. Panel A shows 36 genes possessing 2- to 39-fold (median of all time points) increased expression upon infection, while 10 genes were 2- to 3-fold down-regulated. Panel B and C illustrate mRNAs being temporally induced or repressed after infection. Panel D reflects dysregulated miRNAs after infection, while panel E shows temporally induced miRNAs. An averaged trace of the expression profile is integrated as a white graph by the acuity software (Panel B, C and E). Underlined mRNAs and miRNAs are addressed in the results section.

To assess the expression patterns of immuno- as well as apoptosis-related genes affected by MAH infection, we first regarded genes showing consistent and at least 2-fold (mean) up- and down-regulation upon infection by both MAH strains. As shown in figure 1 A, this analysis revealed 36 genes possessing 2- to 39-fold (median of all time points) increased expression upon infection, while 10 mRNAs were 2- to 3-fold down-regulated. To provide a more in depth insight into the data, mRNAs showing at least 2-fold up- or down-regulation over all time points are summarised in table 1, including those showing expression signals in at least 4 out of 6 samples. Since we did not observe any systematic and strain specific difference in the MDM response to MAH infection, further analysis was performed considering the entire data in a time specific manner. Genes showing significantly differential expression regarding all time points after infection were identified by the analysis of variance (ANOVA). Hereby, we were able to identify two distinct groups of genes showing an initial response of either increased (figure 1 B) or decreased (figure 1 C) expression compared to the non-infected controls, respectively. These expression patterns presumably reflected a temporal regulation of early or late responsive genes in the context of MAH infection.

**Table 1:** Differentially expressed protein coding genes after MAH infection.

Gene Symbol	Gene Desciption	Log 2 Ratio*
INHBA	inhibin, beta A	5.3
CCL1	chemokine (C-C motif) ligand 1	5.1
IL1B	interleukin 1, beta	4.5
VEGFC	vascular endothelial growth factor C	4.0
MMP3	matrix metallopeptidase 3 (stromelysin 1, progelatinase)	3.7
SLAMF1	signaling lymphocytic activation molecule family member 1	3.6
CCR7	chemokine (C-C motif) receptor 7	3.3
MMP1	matrix metallopeptidase 1 (interstitial collagenase)	3.3
TNFAIP6	tumor necrosis factor, alpha-induced protein 6	3.3
TNIP3	ABIN-3, LIND; TNFAIP3 interacting protein 3	3.2
CCL20	chemokine (C-C motif) ligand 20; K05514 C-C motif chemokine, other	3.2
IL7R	interleukin 7 receptor; K05072 interleukin 7 receptor	3.0
PROCR	protein C receptor, endothelial; K06557 protein C receptor, endothelial (EPCR)	3.0
PDGFB	platelet-derived growth factor beta polypeptide (simian sarcoma viral (v-sis) oncogene homolog)	2.9
CSF2	colony stimulating factor 2 (granulocyte-macrophage)	2.8
TNF	tumor necrosis factor	2.8
CCL3-CCL3L1-CCL3L3	chemokine (C-C motif) ligand 3 - chemokine (C-C motif) ligand 3-like 1 - chemokine (C-C motif) ligand 3-like 3	2.5
IL8	interleukin 8; K10030 interleukin 8	2.4
IL3RA	IL3RA, CD123, IL3R, IL3RAY, IL3RX, IL3RY, MGC34174, hIL-3Ra; interleukin 3 receptor, alpha (low affinity	2.4
BMP6	BMP6, VGR, VGR1; bone morphogenetic protein 6; K04663 bone morphogenetic protein 5/6/7/8	2.4
MSC	musculin (activated B-cell factor-1)	2.3
CCL4-CCL4L2-CCL4L1	chemokine (C-C motif) ligand 4 - chemokine (C-C motif) ligand 4-like 2 - chemokine (C-C motif) ligand 4-like 1	2.3
TM4SF1	transmembrane 4 L six family member 1	2.3
TNFRSF9	tumor necrosis factor receptor superfamily, member 9	2.3
MRC1	mannose receptor, C type 1	2.3
LAMB3	laminin, beta 3; K06244 laminin, beta 3	2.2
TNFRSF19	tumor necrosis factor receptor superfamily, member 19	2.2
CCL5	RANTES, chemokine (C-C motif) ligand 5	2.1
CHST2	GST2, carbohydrate (N-acetylglucosamine-6-O) sulfotransferase 2	2.0
ETS2	v-ets erythroblastosis virus E26 oncogene homolog 2 (avian	2.0
TNFSF9	CD137L; tumor necrosis factor (ligand) superfamily, member 9	2.0
CCL18	AMAC1, MIP-4, chemokine (C-C motif) ligand 18 (pulmonary and activation-regulated)	2.0
PTGS2	COX2, prostaglandin-endoperoxide synthase 2 (prostaglandin G/H synthase and cyclooxygenase)	1.9
IL10	interleukin 10	1.9
SOCS3	suppressor of cytokine signaling 3	1.9
SERPINB2	serpin peptidase inhibitor, clade B (ovalbumin)	1.8
SERPINE1	PAI, serpin peptidase inhibitor, clade E (nexin, plasminogen activator inhibitor type 1), member 1	1.8
TIMP1	metallopeptidase inhibitor 1	1.8
BTG1	B-cell translocation gene 1, anti-proliferative	1.8
SOD2	superoxide dismutase 2, mitochondrial	1.8
CD14	CD14 molecule	1.7
MMP10	matrix metallopeptidase 10 (stromelysin 2)	1.5
PLAUR	plasminogen activator, urokinase receptor	1.5
NFKBIA	nuclear factor of kappa light polypeptide gene enhancer in B-cells inhibitor, alpha	1.4
CD40	TNFRSF5, CD40 molecule, TNF receptor superfamily member 5	1.3
MMP14	matrix metallopeptidase 14 (membrane-inserted)	1.3
IRF1	interferon regulatory factor 1	1.3
BIRC3	AIP, baculoviral IAP repeat-containing 3	1.2
ICAM1	CD54, intercellular adhesion molecule 1	1.2
CD44	CD44 molecule	1.1
CDKN1B	cyclin-dependent kinase inhibitor 1B (p27, Kip1)	1.1
BATF	basic leucine zipper transcription factor, ATF-like; K09034 ATF-like basic leucine zipper transcriptional factor	1.1
CSF2RA	GMCSFR, colony stimulating factor 2 receptor, alpha, low-affinity (granulocyte-macrophage)	1.1
FCGR2A-FCGR2B-FCGR2C	Fc fragment of IgG, low affinity IIa, receptor (CD32) - Fc fragment of IgG, low affinity IIb, receptor (CD32) - Fc fragment of IgG, low affinity IIc, receptor for (CD32) (gene/pseudogene)	1.1
CD82	KAI1, CD82 molecule, kangai 1	1.0
ITGA5	CD49e, integrin, alpha 5 (fibronectin receptor, alpha polypeptide)	1.0
CXCL10	chemokine (C-X-C motif) ligand 10	1.0
QSOX1	quiescin Q6 sulfhydryl oxidase 1	1.0
PIAS1	protein inhibitor of activated STAT, 1	−1.0
PRKCB	protein kinase C, beta	−1.0
SMAD2	MADH2, SMAD family member 2, mothers against DPP 2/3	−1.0
PARP1	poly (ADP-ribose) polymerase 1	−1.0
CD9	MIC3, CD9 molecule	−1.0
IL18BP	interleukin 18 binding protein	−1.1
NFATC3	nuclear factor of activated T-cells, cytoplasmic, calcineurin-dependent 3	−1.1
CD52	CD52 molecule	−1.1
LGALS3	GALBP, lectin, galactoside-binding, soluble, 3	−1.1
CCNE1	cyclin E1	−1.2
STMN1	stathmin 1	−1.6
LTA4H	leukotriene A4 hydrolase	−1.7
CD36	CD36 molecule	−1.7

*Binary log ratio of increased or decreased gene expression according to microarray analysis of MAH infected macrophages compared to non-infected controls. At least 2-fold (corresponding to Log 2 ratios of ≥1 or ≤−1) altered expression of median of values for 6, 24 and 48 h, as well as MAH 10091/06 and MAH 104 was considered.

The identified clusters presented above, which were either dysregulated among all time points (figure 1 A) or possessed a temporal dysregulation (figure 1 B and C) were chosen for pathway analysis using the Cytoscape [22] plug-in ClueGO [23]. For this purpose, we added also genes to the cluster shown in figure 1 A that did not exhibit consistent expression on all microarrays. Pathways showing functional enrichment are shown in table 2 together with involved and differentially expressed genes. Affected pathways reflect many well described functional processes involved in mycobacterial interference with the host response. It is known that pathogenic mycobacteria promote intracellular survival in macrophages by affecting phagosome-lysosome-fusion, antibacterial responses involving for example Ca^2+^ signalling, PI3K, JAK/STAT, MAPK and TLR pathways as well as apoptosis. The microarray data presented in this study indicated regulated expression of various genes involved in signalling pathways mentioned above.

**Table 2:** Distribution of differentially expressed genes among biochemical pathways.

Functionally enriched Pathways*	% of Genes**	Associated Genes***
Fc Epsilon Receptor I Signaling in Mast Cells	20	BTK, JUN, LYN, MAPK3, PRKCB, SYK
Fc epsilon RI signaling pathway	12	BTK, CSF2, LYN, MAP2K3, MAPK3, PLCG2, PRKCB, SYK, TNF
Complement and coagulation cascades	9	C1QA, C3AR1, CD46, PLAU, PLAUR, SERPINE1
Toll-Like Receptor Pathway	19	CD14, JUN, MAP2K3, MYD88, NFKB1, NFKBIA, TLR2
Toll-like receptor signaling pathway	16	CCL5, CD14, CD40, CXCL10, IL1B, IL8, JUN, MAP2K3, MAP3K8, MAPK3, MYD88, NFKB1, NFKBIA, STAT1, TLR2, TNF
Apoptosis	14	BID, BIRC3, CASP3, CASP6, CFLAR, FAS, IL1B, IL3RA, MYD88, NFKB1, NFKBIA, TNF
Apoptotic Signaling in Response to DNA Damage	24	BID, CASP3, CASP6, PARP1, STAT1
Caspase Cascade in Apoptosis	17	BIRC3, CASP3, CASP6, PARP1
FAS signaling pathway (CD95)	20	CASP3, CASP6, CFLAR, FAS, JUN, PARP1
p53 signaling pathway	10	BID, CASP3, CCNE1, CD82, CDKN1A, FAS, SERPINE1
ATM Signaling Pathway	21	BRCA1, JUN, NFKB1, NFKBIA
Free Radical Induced Apoptosis	44	IL8, NFKB1, SOD1, TNF
Induction of apoptosis through DR3 and DR4/5 Death Receptors	21	BID, CASP3, CASP6, CFLAR, NFKB1, NFKBIA
Influence of Ras and Rho proteins on G1 to S Transition	25	CCNE1, CDKN1B, MAPK3, NFKB1, NFKBIA, RHOA
Role of Mitochondria in Apoptotic Signaling	19	BID, BIRC3, CASP3, CASP6
NF-kB Signaling Pathway	18	MYD88, NFKB1, NFKBIA, TNF
NFkB activation by Nontypeable Hemophilus influenzae	33	IL1B, IL8, MAP2K3, MYD88, NFKB1, NFKBIA, TLR2, TNF
Cytokine-cytokine receptor interaction	11	CCL1, CCL18, CCL20, CCL22, CCL5, CCR1, CCR5, CCR7, CD40, CSF2, CSF2RA, CXCL10, FAS, IFNGR1, IL10, IL1B, IL3RA, IL7R, IL8, INHBA, PDGFB, TNF, TNFRSF19, TNFRSF9, TNFSF13, TNFSF13B, TNFSF9, VEGFB, VEGFC
Cytokines and Inflammatory Response	15	CSF2, IL10, IL8, TNF
Dendritic cells in regulating TH1 and TH2 Development	19	CD40, CSF2, IL10, TLR2
IL-2 Receptor Beta Chain in T cell Activation	15	CFLAR, FAS, MAPK3, SOCS3, SYK
T cell receptor signaling pathway	10	CSF2, IL10, JUN, MAP3K8, NFATC3, NFKB1, NFKBIA, RHOA, TNF
Cadmium induces DNA synthesis and proliferation in macrophages	36	JUN, MAPK3, NFKB1, NFKBIA, TNF
Selective expression of chemokine receptors during T-cell polarization	19	CCR1, CCR5, CCR7, CSF2, IFNGR1
B cell receptor signaling pathway	14	BTK, JUN, LYN, NFATC3, NFKB1, NFKBIA, PLCG2, PRKCB, SYK
Natural killer cell mediated cytotoxicity	9	BID, CASP3, CSF2, FAS, ICAM1, IFNGR1, MAPK3, NFATC3, PLCG2, PRKCB, SYK, TNF
fMLP induced chemokine gene expression in HMC-1 cells	16	MAP2K3, MAPK3, NFKB1, NFKBIA
TNF/Stress Related Signaling	21	JUN, MAP2K3, NFKB1, NFKBIA, TNF
TNFR1 Signaling Pathway	14	CASP3, JUN, PARP1, TNF
The 4-1BB-dependent immune response	29	JUN, NFKB1, NFKBIA, TNFRSF9, TNFSF9
Mechanism of Gene Regulation by Peroxisome Proliferators via PPARa(alpha)	12	HSP90AA1, JUN, MAPK3, NFKBIA, TNF
Signal transduction through IL1R	24	IL1B, JUN, MAP2K3, MYD88, NFKB1, NFKBIA, TNF
MAPKinase Signaling Pathway	10	JUN, MAP2K3, MAP3K8, MAPK3, MAPK6, NFKB1, NFKBIA, STAT1
ECM-receptor interaction	9	CD36, CD44, CD47, ITGA5, ITGA6, ITGB3, ITGB5, LAMB3
Epithelial cell signaling in Helicobacter pylori infection	12	CASP3, CCL5, IL8, JUN, LYN, NFKB1, NFKBIA, PLCG2
Hematopoietic cell lineage	17	CD14, CD36, CD44, CD9, CSF2, CSF2RA, FCGR1A, IL1B, IL3RA, IL7R, ITGA5, ITGA6, ITGB3, MME, TNF
Chaperones modulate interferon Signaling Pathway	24	IFNGR1, NFKB1, NFKBIA, TNF
Alzheimer's disease	18	C1QA, CASP3, IL1B, MME, TNF

*Pathways containing at least 4 differentially expressed genes (or 8%). Underlined pathways are referred to in the results section.

**Percentage of the differentially expressed genes which share a pathway membership with regard to all pathway-associated genes according to Reactome/Biocarta and Kegg pathway databases implemented in Cytoscape 2.7.0.

***Differentially expressed genes with shared pathway memberships. Differentially expressed genes with at least 1.5-fold altered expression compared to non-infected control cells and having valid expression signals in at least 4 out of 6 microarray experiments were considered for pathway mapping. Genes showing temporal expression pattern, as depicted in figure 1 B and C, were also included in the analysis.

An initial process with a potential role in affecting intracellular survival is the internalisation of mycobacteria by macrophages. This is mediated by phagocytic receptors including Fc-gamma receptor (FCGR), complement receptors (CR) of the integrin family (ITG) and mannose receptors (MRC). Indications of involvement of those signalling events are reflected in table 2. Accordingly, differential expression of FCGR1A and FCGR2 was detected as shown in figure 1 B, while expression signals for FCER2 were below detection limit of the applied method. More precisely, FCGR1A showed more than 3-fold reduced expression levels for both MAH strains at 6 h p.i. Furthermore, most members of the integrin family were down-regulated at the same time (ITGAM, ITGAX, ITGB2, data not shown). MRC1 showed 2- to 5-fold increased expression at 24 and 48 h p.i. (table 1).

After internalisation, phagosomal maturation events take place, which involve fusion of various vesicles and formation of phago-lysosomes. Phagosomal markers include lysosome-associated membrane proteins LAMP1 and LAMP2 and acid hydrolases like cathepsin D (CTDS) and sytaxin 3 (STX3). Pathogenic mycobacteria further have been shown to interfere with Ca^2+^ and PI3K signalling pathways, which are essential for phagosomal maturation and involve calmodulin (CALM2) activation. Our data reflected a consistent 2-fold (median) down-regulation of CALM2 at all time points, markedly reduced STX3 expression at 48 h p.i. (data not shown) and decreased CTSD (Fig. 1C) mRNA at 6 h p.i. LAMP1 and LAMP2 expression was down-regulated at 6 h or 48 h p.i., respectively (data not shown).

When invading bacteria are detected by the host, the activation of signalling pathways like JAK/STAT and MAPK pathways lead to a release of pro-inflammatory cytokines. JAK/STAT signalling is induced after binding of IFNG to its receptors (IFNGRs), whereas SOCS directly bind JAKs and inactivate JAK/STAT signalling. Accordingly, in our study IFNGR1 expression was 1.7-fold reduced at 6 h p.i. (figure 1 B). Additionally, we observed an initial 3.6-fold reduced STAT1 expression early after infection (6 h p.i.), while expression was up to 2-fold increased after 24 h and 48 h (figure 1 B). Further, our data reflected an averaged 3.8-fold up-regulation of SOCS3 at all time points (table 1). In the context of interfering with MAPK signalling, we observed a regulation of PLCG2 as illustrated in figure 1 B. A pronounced down-regulation of PLCG2 was observed, showing 2.4-fold reduced expression early after MAH infection (6 h p.i.). TLRs and CD40 (TNFRSF5) were reported not to be implicated in the uptake of *M. tuberculosis* but mediate signalling events upon activation [24]. TLR activation typically induces NFκB and MAPK pathways resulting in the production of pro-inflammatory cytokines. According to our microarray study, at 48 h p.i. TLR4, TRAF6 and MyD88 were transcriptionally down-regulated upon *M. avium* infection (data not shown). Furthermore, TLR2 was up-regulated at 24 and 48 h p.i. (figure 1 B), while CD40 showed 2.6-fold (mean) increased expression at all time points (figure 1A).

As mentioned above, the activation of macrophages upon mycobacterial infection leads to an innate immune response. Innate immunity is linked to production of pro-inflammatory cytokines. Furthermore, in response to pathogens macrophages are functionally polarised and can be broadly classified into two groups: M1 and M2. Put simply, M1 macrophages show microbicidal and inflammatory character, while M2 macrophages are immunomodulators and poorly microbicidial [25]. Both possess characteristic gene expression profiles of e.g. ILs, TGFB, VEGF, MMPs, CXCL10 or TNF. These genes also show regulated expression in the presented study as follows. Our microarray data showed strong up-regulation of IL1B (23-fold), IL8 (5.3-fold) and IL10 (3.7-fold) at all time points and in both MAH strains (figure 1 A, table 1). IL7R showed an identical pattern of averaged 8-fold up-regulation (table 1), except at 48 h p.i., when the expression value for MAH 104 was not detected in the microarray experiments. IL3RA showed time-dependent regulation with reduced expression at 6 h p.i. and up-regulation at 24 h and 48 h (figure 1 B, table 1). IL18BP was down-regulated at all time points and in both MAH strains (figure 1 A, table 1). IL2RA, IL6 and IL1R1 and IL12A were exclusively detectable in MAH 10091/06 infected MDMs and showed consistent up-regulation (data not shown). This is also true for IL15 at 6 h and 24 h after infection (data not shown). In the context of macrophage polarisation, we further observed a 14-fold induced expression of VEGFC, one of several isoforms of VEGF, and a 2.3- to 16-fold induced expression of several MMPs (MMP1, MMP3, MMP10, and MMP14) averaged over all time points and MAH strains (table 1, figure 1 A). The described patterns point to a M2 polaraisation. However, CXCL10 was 2-fold up-regulated at all time points (mean value, table 1) and TNF showed 6.7-fold elevated transcript levels at all time points (mean value, figure 1 A, table 1) both representing M1 polarisation of macrophages. TGFB1 showed induced expression after 24 h p.i. (figure 1 B).

Many pathogens influence host apoptotic pathways [26]. Various indications for altered apoptotic signalling are reflected by the microarray data presented here. Stimulation of Ca^2+^ signalling facilitates apoptosis by increasing the permeability of mitochondrial membranes, thereby promoting the release of pro-apoptotic factors such as cytochrome C. The microarray data of CALM2, STX3 and CTSD regulation was already mentioned in the context of Ca^2+^ signalling and phagosome maturation. Our data (table 2) further reflected the extrinsic pathway, which involves activation of death receptors and CASP8/10 and CASP3. In the microarray data CASP3, 6 and 7 were down-regulated, with most pronounced regulation at 48 h p.i. (figure 1 C or data not shown).

Down-regulation of caspases in infected MDMs suggested the ability of MAH strains to inhibit apoptosis similar to other virulent mycobacteria.

### Identification of differentially expressed miRNAs and regulative networks of MAH infection deduced from integrated miRNA-mRNA microarray analysis

Contrary to a general induction of mRNA expression in MAH infected cells, many of differentially regulated miRNAs showed decreased expression upon MAH infection compared to the controls (figure 1 D). However, miR-155, miR-146a, and miR-886-5p were consistently at least 1.4-fold up-regulated upon MAH infection. In the general context of pathogen interaction with immune cells, this is the first study reporting miR-886-5p induction at 48 h p.i. Also, a temporal induction of miRNAs was found after microarray analysis (figure 1 E), which occurred mainly after 24 h and persisted until the end of the infection experiments. For example, miR-29a was induced after 48 h possessing 1.5-fold increased expression compared to the non-infected control. Interestingly, let-7e was reported to be involved in macrophage response to LPS [27] and miR-146b is known to target TLR signalling [18], and both were 1.8- and 1.7-fold up-regulated after 48 h, respectively (figure 1 E).

**Figure 2:**
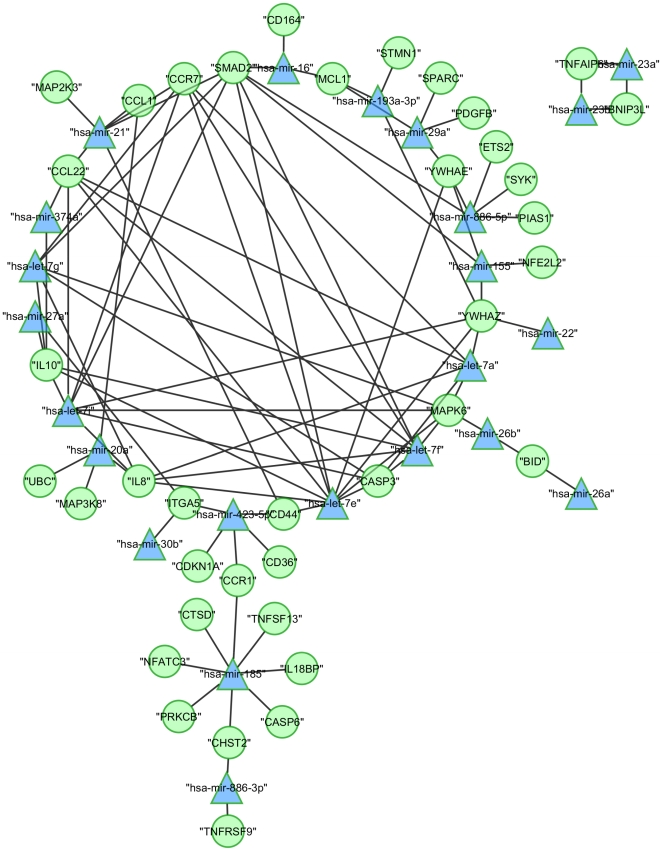
Regulatory network of MAH infected MDMs deduced from integrated analysis of miRNA-mRNA microarray data. Negatively correlated miRNA-mRNA interactions were visualised as a network using Cytoscape. This network gives for the first time an theoretical outline of the concerted action of regulating miRNAs (blue triangles) and their potential target mRNAs (green circles) in mycobacterial infection of human macrophages.

As described in the previous section, our data comprised many mRNAs and miRNAs possessing a negatively correlated expression. Some of these correlations are validated and published interactions between miRNAs and their target mRNAs, however many potential interactions are still to be discovered. Our aim was to create a theoretical concept of an interacting network based on linking miRNA and mRNA expression data to target prediction. To facilitate the prediction of possible interactions from comprehensive data presented here, we have employed the integrative analysis of target prediction as well as miRNA and mRNA expression by using the tool MAGIA [19]. We first predicted targets of all detected miRNAs using the combined PITA and Target Scan analysis implemented in the MAGIA query (not considering the expression data). The hereby identified target list was initially subjected to pathway analysis using the Cytoscape Plug-In ClueGO (table S1). It was intriguing to find out that predicted targets of miRNAs were enriched in pathways being mainly involved in cell death/apoptosis and immune signalling. This very well reflected affected cellular processes determined by mRNA expression analysis as described above (table 2). Subsequently, we performed the MAGIA analysis considering the expression data of dysregulated mRNAs showing at least 2-fold altered expression or a temporal expression pattern (figure 1 A–C) and considering all detected miRNAs of corresponding samples, respectively. Table S2 summarises miRNAs and their predicted target genes including the calculated correlation coefficients (Pearson r). Negatively correlated miRNA-mRNA interactions were visualised as a network using Cytoscape (figure 2). This network gives for the first time a theoretical outline of the concerted action of regulating miRNAs and their potential target mRNAs in mycobacterial infection of macrophages.

To give an example, according to this analysis CD44 is targeted by let-7e and respective expression profiles are negatively correlated (r = −0.85). Reflecting CD44 expression, we observed a 3.2- or 4-fold induction at 6 h p.i. considering MAH 10091/06 and 104, respectively (figure 1 C, table 1). In MAH 10091/06 infected cells a gradual decline of CD44 expression is observed towards the later time points, while after infection with MAH 104 a decline in expression is observed from 6 h to 24 h p.i. By contrast, let-7e expression is increasingly induced during the course of MAH infection (figure 1 E). In order to predict functional relations and to simplify the vast number of interactions shown in figure 2, identified target genes were aligned to pathway databases as described above. Table 3 summarises the determined interactions and potentially affected pathways using ClueGO default settings. CASP3, CASP6 and BID that are involved in induction of apoptosis through death receptors were identified as potential targets of let-7, miR-26, miR-30 and miR-185. IL10, IL8 and MAP2K3 being involved in the NFκB mediated inflammatory response were predicted to be regulated by let-7 and miR-20a.

The complex predicted interactions of miRNAs targeting dysregulated genes (figure 2) puts further emphasis on our hypothesis that expression and regulation of miRNAs upon MAH infection orchestrates mechanisms promoting cell survival. In order to perform a more detailed analysis of the regulation of cell fate, we specifically regarded apoptosis related genes in further experiments.

**Table 3:** Functional enrichment of pathways deduced from integrated miRNA-mRNA analysis.

Functionally enriched Pathways*	Potential Target Genes**	Potential miRNA regulators***	% of Genes****
Cytokines and Inflammatory Response	IL10, IL8	hsa-let-7e, hsa-let-7f, hsa-let-7g, hsa-let-7i, hsa-mir-27a, hsa-mir-374a, hsa-let-7a, hsa-mir-20a	8
NFkB activation by Nontypeable Hemophilus influenzae	IL8, MAP2K3	hsa-let-7i, hsa-mir-20a, hsa-let-7a, hsa-let-7ghsa-let-7f, hsa-let-7e, hsa-mir-21	8
Caspase Cascade in Apoptosis	CASP3, CASP6	hsa-let-7i, hsa-let-7e, hsa-let-7f, hsa-let-7g, hsa-let-7a, hsa-mir-30b, hsa-mir-30c, hsa-mir-185	9
Induction of apoptosis through DR3 and DR4/5 Death Receptors	BID, CASP3, CASP6	hsa-mir-26b, hsa-mir-26a, hsa-let-7i, hsa-let-7e, hsa-let-7f, hsa-let-7g, hsa-let-7a, hsa-mir-30b, hsa-mir-30c, hsa-mir-185	11
FAS signaling pathway ( CD95 )	CASP3, CASP6	hsa-let-7i, hsa-let-7e, hsa-let-7f, hsa-let-7g, hsa-let-7a, hsa-mir-30b, hsa-mir-30c, hsa-mir-185	7
TSP-1 Induced Apoptosis in Microvascular Endothelial Cell	CASP3, CD36	hsa-let-7i, hsa-let-7e, hsa-let-7f, hsa-let-7g, hsa-let-7a, hsa-mir-30b, hsa-mir-30c, hsa-mir-423-5p	29
Fc Epsilon Receptor I Signaling in Mast Cells	PRKCB, SYK	hsa-mir-185, hsa-mir-886-5p	7
Selective expression of chemokine receptors during T-cell polarization	CCR1, CCR7	hsa-mir-185, hsa-mir-423-5p, hsa-let-7g, hsa-mir-21, hsa-let-7f, hsa-let-7i, hsa-let-7e, hsa-let-7a, hsa-mir-423-5p	8

*Pathways containing at least 2 differentially expressed genes (or 4%).

**Predicted Target genes showing negatively correlated expression compared with miRNA expression data. Differentially expressed genes with at least 2-fold altered or temporal expression and regarding expression data of each time point and MAH strain were considered (listed in figure A–C).

***miRNAs predicted to target differentially expressed genes with negatively correlated expression data. Each miRNA is predicted to target at least one of the listed genes in column 2.

****Percentage of the differentially expressed genes which share a pathway membership with regard to all associated genes according to Reactome/Biocarta and Kegg pathway databases implemented in Cytoscape 2.7.0.

### MAH strains are able to inhibit apoptosis

For focusing our investigation on identified pathways and to validate microarray data, we performed qRT-PCR experiments for mRNAs as well as miRNAs. The individual response of MDMs from three independent donors was studied by additionally implying another MAH strain (*M. avium hominissuis* 2514) that was originally isolated from water as well as including the non-pathogenic *E. coli* K12 as a control. Since *M. avium hominissuis* occurs ubiquitously in soil and water, MAH 2514 was included to evaluate potential differences between patient and environmental isolates.

**Figure 3:**
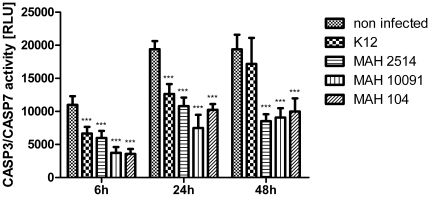
Temporal CASP3/CASP7 activity upon MAH infection of MDMs. MDMs were infected with bacteria and the luminescence signal (relative light units, RLU) proportional to the activity of CASP3/CASP7 was measured. Columns represent the mean of quintuplicate measurements while error bars show the standard deviation. Asterisks indicate statistical significance according to paired t test (***: P<0.001).

**Figure 4:**
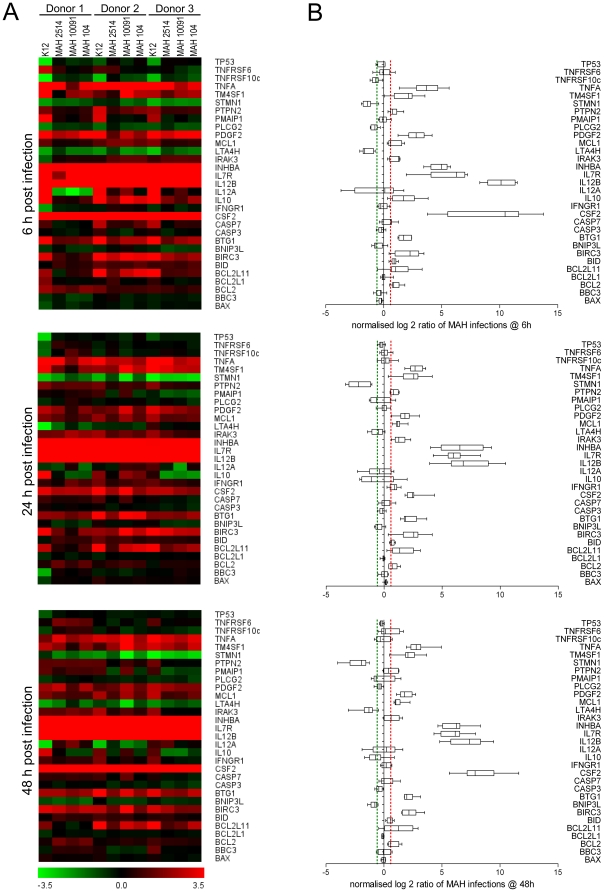
mRNA expression data of MAH infected MDMs after qRT-PCR analysis. Columns in panel A represent the individual expression of MDMs from three different human donors (Donor 1–3) infected with MAH strains 2514, 10091/06 and 104 as well as *E. coli* K12 as a control. Colours represent log 2 ratios of the means of triplicate measurements of infected cells versus the non-infected control according to the scales shown below. The box plots in panel B include the data obtained from all three infection experiments considering all three MAH strains. Log 2 ratios below −0.585 and above 0.585 (corresponding to 1.5-fold change) are indicated by the green and red dashed lines and were considered to reflect differential expression.

In order to assess the ability of MAH strains to inhibit apoptosis, CASP3/CASP7 assays were performed. As shown in figure 3, at 6 and 24 h p.i. significantly decreased caspase activity was observed in all infected samples compared to the non-infected controls. However, at 48 h p.i. MDMs infected with all three MAH strains showed significantly decreased caspase activity compared to the non-infected as well as *E. coli* K12 stimulated controls (P<0.001, paired t test), while no significant difference was detected between the latter. Accordingly, lower caspase expression was detected in qRT-PCR experiments (figure 4 A). Based on the observation that the employed MAH strains are able to inhibit apoptosis, we considered mainly genes in the qRT-PCR experiments being relevant to apoptosis and cytokine-cytokine receptor interaction. For visualisation of the comprehensive and individual expression data of selected genes, heat maps of log 2 ratios of infected samples and non-treated controls were created for each point in time. As shown in figure 4 A, MAH infections caused a unique response of MDMs compared to the *E. coli* K12 stimulation. The detailed expression analysis of selected genes showed on the one hand some donor specific differences in response to the MAH infection. For example, all MAH strains caused increased expression of IFNGR1 in donor 2 right after 6 h compared to the other samples (P<0.002, unpaired t test). The up-regulation of IFNGR1 appeared after 24 h in all samples followed by decreased expression after 48 h only in donor 3. Accordingly, we observed sustained and consistent induction of TNF receptor superfamily (TNFRSF) 6 and TNFRSF10C after 24 h by all MAH strains in donor 1 compared to the other donors (figure 4).

On the other hand, a mostly consistent reaction of each donor to all three MAH strains was investigated, indicating marginal differences between MAH strains in terms of immunogenicity. While e.g. IL12B expression was increased right after infection and throughout the entire experiment, IL10 and IL12A showed lower expression in early infection stage compared to *E. coli* K12 stimulated MDMs (P<0.005, unpaired t test). PTPN2 a member of the protein tyrosine phosphatase family that was reported to regulate IFNG induced cytokine signalling [28] exhibited also significantly lower expression after MAH exposition particularly at 6 h p.i. (P<0.004, unpaired t test) but also up to 48 h (P<0.04, unpaired t test) compared to *E. coli* K12. In order to investigate the observed PLCG2 down-regulation in our microarray experiments in more detail, we included also PLCG2 in our qRT-PCR experiments. However, PLCG2 expression was decreased at 6 h p.i. in all samples possessing no statistically significant difference between samples (figure 4 A and B). In late stages of infection (24 and 48 h p.i.) the pro-apototic gene BNIP3L was down-regulated in response to all MAH infections compared to *E. coli* K12 stimulation (P<0.05, unpaired t test). Another apoptotic activator BCL2L11 was significantly down-regulated right after MAH infection and showed sustained and decreased expression along the entire experiment compared to *E. coli* K12 (P≤0.01, unpaired t test). On the other hand, in later infection stages BCL2L1, a pro-survival member of the BCL2 family, showed consistently decreased expression in *E. coli* K12 compared to MAH infected MDMs (P<0.015, unpaired t test). Surprisingly, while *E. coli* K12 induced pronounced up-regulation of the pro-apoptotic gene PMAIP1 at 6 h p.i. all three donors responded on average with 8 times lower PMAIP1 expression to MAH infection (P<0.0001, unpaired t test). At this point in time MAH infection caused a balanced expression of PMAIP1 in donor 1 compared to the non-infected control, while the other two donors showed even down-regulation of PMAIP1 upon MAH infection. Another BCL2 family member, BAX, acts as an apoptotic activator by effecting cytochrome C release. After 48 h it was significantly up-regulated in all *E. coli* K12 stimulated samples while showing lower expression in all MAH infected MDMs (P<0.001, unpaired t test). With special interest we observed significant up-regulation of the negative regulator of TLR signalling IRAK3 in all MAH infected MDMs compared to *E. coli* K12 stimulation at 6 h p.i. (P<0.05, unpaired t test). Generally, we did not observe pronounced MAH strain specific differences in our infection experiments. But we realised with particular interest the consistent dysregulation of leukotriene A4 hydrolase gene (LTA4H) at 6 h p.i. with the environmental isolate MAH 2514 compared to the two other strains, which were isolated from human patients. MAH 2514 caused on average double expression values in all donors compared to the other strains (P<0.0001, unpaired t test). But based on the observation that the investigated MAH strains caused broadly similar MDM response to infection and to simplify the data, we decided to consider the medians of log 2 ratios of all MAH strains for further analysis. The box plots in figure 4 B include the data obtained from all three infection experiments considering only the three MAH strains. Log 2 ratios below −0.585 and above 0.585 (corresponding to 1.5-fold change) were considered to be differentially expressed. We applied the tool KegArray [29] for pathway analysis of dysregulated genes using the calculated medians of log 2 ratios (figure 4 B) of up- or down-regulated genes. This analysis once again showed that MAH infection induced mainly genes directing survival e.g. IRAK3, BIRC3 or BCL2 and underlining the up-regulation of NFκB-transcripts e.g. TNF, IL12, BCL2 or BIRC3. Taken together, six genes (CSF2, IL7R, IL12B, INHBA, PDGFB and TNF) were identified to be dysregulated across MAH infection being related to the “cytokine cytokine-receptor interaction” category of KEGG. IRAK3, BIRC3 and TNF were dysregulated throughout the entire infection and were classified into the apoptosis pathway.

### MAH infection of MDMs causes concomitant up-regulation of let-7e and miR-29a targeting key caspases CASP3 and 7

**Figure 5:**
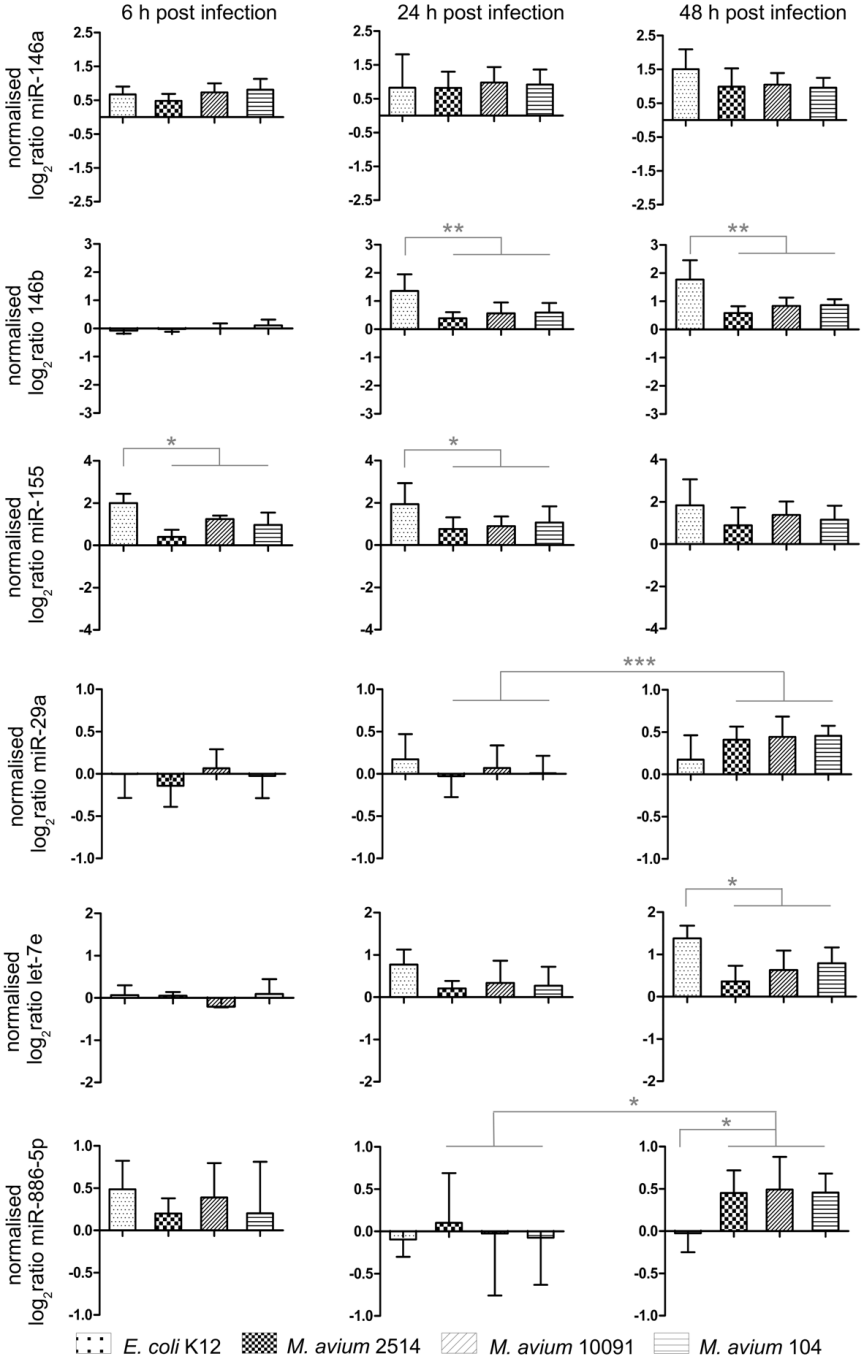
miRNA expression data of MAH infected MDMs after miRNA specific qRT-PCR analysis. The columns show the mean expression of distinct miRNAs from all three donors each measured in triplicates while error bars show the standard deviation. The calculated log 2 ratios of means of all donors relating to each MAH strain and *E. coli* K12 are shown, respectively. Asterisks indicate statistical significance according to unpaired t test (*: P<0.05; **: P<0.01; ***: P<0.001).

For validation of miRNA microarrays and to investigate both the donor and MAH strain specific progress of the infection, we have applied a miRNA specific qRT-PCR approach, called miR-Q [30]. The individual response of MDMs isolated from different donors was studied using the same RNA samples as described for the mRNA qRT-PCRs by also including the MAH strain 2514 as well as the non-pathogenic *E. coli* K12 as a control. For this purpose, we mainly considered temporally induced miRNAs. Figure 5 shows the expression of selected miRNAs after infection with MAH strains as well as *E. coli* K12. Since the expression of distinct miRNAs differed only slightly between the three donors (indicated by the standard deviations in figure 5), we decided to calculate the log 2 ratios of means of all donors relating to each MAH strain and *E. coli* K12, respectively. While miR-146a was induced right after infection and showed slightly increased expression up to 48 h p.i., no significant differences were observed dependent on the infectious agent. On the other hand, miR-146b was first up-regulated after 24 h and showed similar expression patterns after 48 h. MAH strains caused significantly lower miR-146b induction (P<0.005, unpaired t test) at both points in time compared to the *E. coli* K12 stimulation. MiR-155 was induced by all bacteria (figure 5); while at 6 and 24 h p.i. *E. coli* K12 stimulation caused a significantly higher expression of miR-155 compared to MAH strains (P<0.04, unpaired t test). Interestingly, MDMs infected with MAH strains showed increased miR-29a expression after 48 h. As shown in figure 5, the *E. coli* K12 stimulated MDMs showed the same miR-29a expression level at 48 h compared to 24 h after infection. MAH strains, however, caused significantly increased expression of miR-29a only at the end of the infection experiment (P≤0.0002, unpaired t test). Progressing course of infection caused a clear induction of let-7e in all samples, but MAH infected MDMs exhibited significantly lower expression compared to the *E. coli* K12 stimulation (P<0.005, unpaired t test). With particular interest we observed significant up-regulation of miR-886-5p only in MAH infected MDMs at 48 h compared to the control (P≤0.0255, unpaired t test). A significant difference between all MAH infected MDMs and *E. coli* K12 stimulation was only observed at this time point (figure 5).

**Figure 6:**
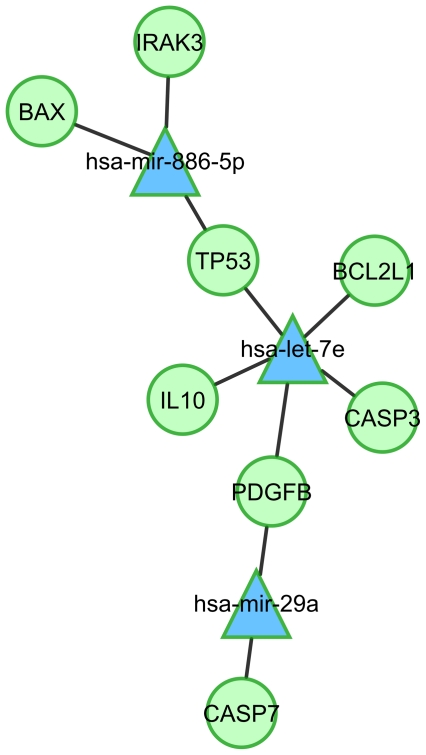
Regulatory network of MAH infected MDMs deduced from integrated analysis of miRNA-mRNA expression after qRT-PCR experiments. Negatively correlated miRNA-mRNA interactions were visualised as a network using Cytoscape. Lines represent predicted interactions considering negatively correlating miRNA (blue triangles) and mRNA (green circles) expression data.

For the integrated miRNA-mRNA-analysis of the qRT-PCR data, we have performed a MAGIA analysis of corresponding log 2 ratios shown in the heat maps in figure 4 A and all log 2 ratios of miRNAs shown in figure 5. Figure 6 shows the Cytoscape network generated from only negatively correlated MAGIA predicted interactions. Network analysis showed IRAK3 and BAX to be two target genes of miR-886-5p both exhibiting significant down-regulation in MAH infection at 48 h compared to 24 h. RNAhybrid analysis [31] of potential interactions between the miRNA and the 3′ UTR revealed three potential target sites with mfe<−20 kcal/mol within the 3′ UTR of IRAK3 and five possible interactions between miR-886-5p and BAX including one significant interaction (P<0.04; data not shown). Consistently, IRAK3 expression was decreased at 48 h after MAH infection significantly compared to 24 h (P<0.05; figure 4 B). Network analysis showed mutual targeting of TP53 by let-7e and miR-886-5p each possessing five possible target sites with mfe<−20 kcal/mol including two significant interactions of let-7e (P<0.033; data not shown) calculated by RNAhybrid. The network demonstrated that the platelet-derived growth factor beta (PDGFB or PDGF2) is mutually targeted by two miRNAs, miR-29a and let-7e being both up-regulated in late stage of MAH infection. Interestingly, MDMs infected with MAH strains exhibited a significant decrease of PDGFB expression after 24 h and 48 h compared to 6 h, respectively (P<0.026, unpaired t test). A more in-depth analysis of the interactions of these miRNAs and the 3′ UTR of PDGFB based on RNAhybrid calculation predicted four possible target sites of let-7e possessing a minimum free energy (mfe) of less than −20 kcal/mol and including one significant site (P<0.05; data not shown). Also one significant site of interaction between PDGFB and miR-29a was calculated possessing an mfe = −24.5 kcal/mol and P≤0.0243 (data not shown). The target site analysis using the RNAhybrid algorithm pointed out five possible interactions between let-7e and the 3′ UTR of IL10, all possessing no significant P values and higher mfe than −20 kcal/mol (data not shown). The calculated network in figure 6 moreover showed an interaction between let-7e and BCL2L1, which showed significantly decreased expression after *E. coli* K12 stimulation compared to the MAH infected MDMs at 24 h as well as 48 h. RNAhybrid analysis also revealed the identification of three target sites of let-7e within the 3′ UTR possessing an mfe<−20 kcal/mol while two were predicted to be significant interactions (P<0.0064 and P<0.037; data not shown).

**Figure 7:**
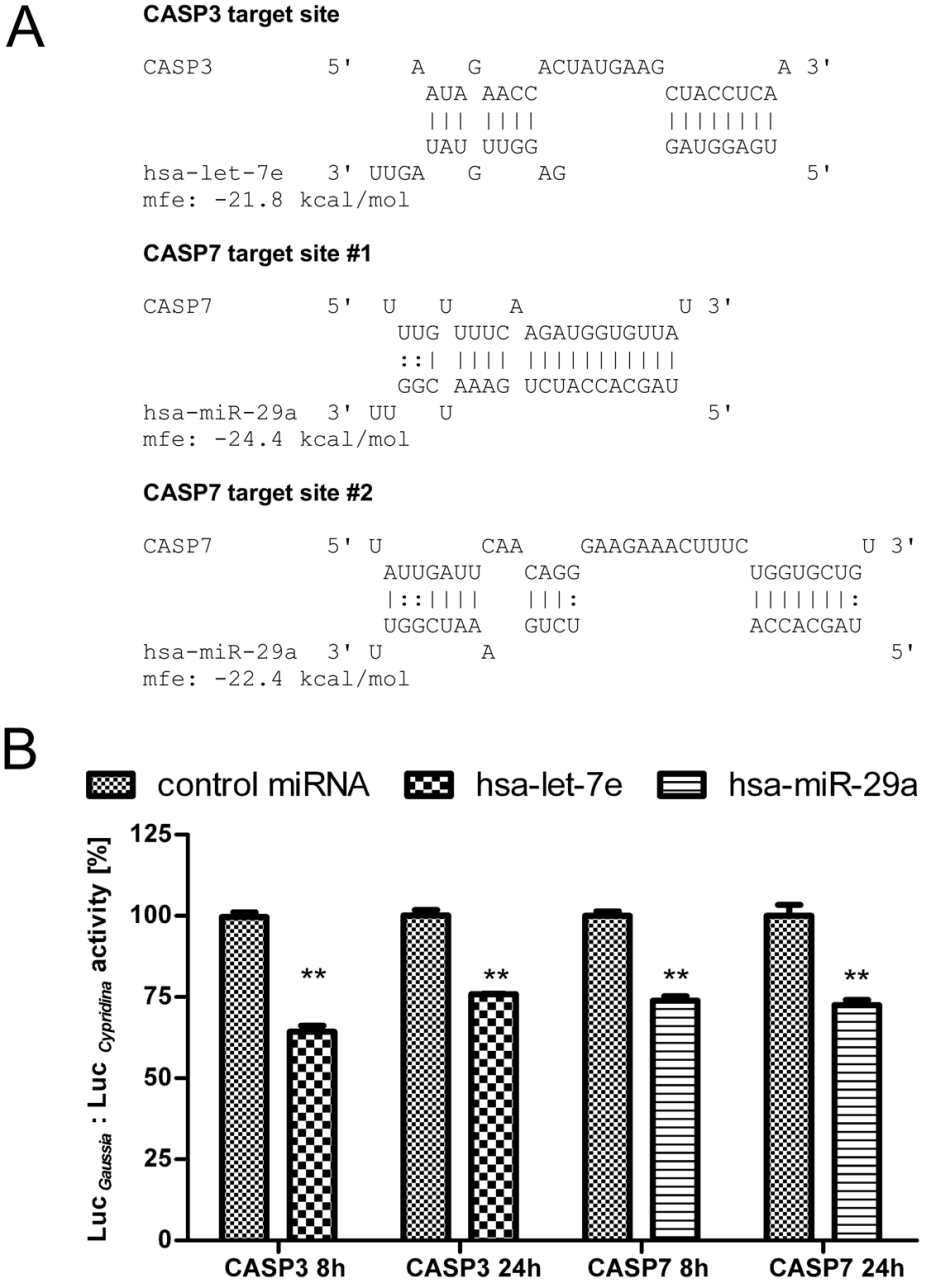
let-7e and miR-29a target CASP3 and CASP7, respectively. Down-regulation of CASP3 and CASP7 by let-7e and miR-29a was verified using reporter gene assays. Panel A: Identified target sites between miRNAs and both caspases were analysed using RNAhybrid. Panel B: HeLa cells were co-transfected with miRNA mimics and plasmids harbouring the 3′ UTR of CASP3 and 7, respectively. Relative luciferase activity (Luc *_Gaussia_* : Luc *_Cypridina_*) was determined respective to the a non-sense miRNA mimics serving as a control. The columns show means of normalised luciferase activity each measured in triplicates while error bars show the standard deviation. Asterisks indicate statistical significance between miRNA treated samples and non-sense miRNA treated controls according to paired t test (**: P<0.01).

Surprisingly, the network generated from the qRT-PCR data revealed CASP3 and CASP7 to be targeted by let-7e and miR-29a both possessing increased expression only in MAH infected MDMs at 48 h compared to 24 h. As shown in figure 7 A, RNAhybrid analysis revealed one potential target site of let-7e with mfe<−20 kcal/mol within the 3′ UTR of CASP3 and two possible target sites of miR-29a within the 3′ UTR of CASP7 (P<0.05). To confirm functional relevance of presented networks and to address the hypothesis that the observed CASP3/7 down-regulation is directed by these two miRNAs, we performed reporter gene assays by fusing the 3′ UTRs of CASP3 and CASP7 into the Gaussia luciferase (Luc *_Gaussia_*), respectively. 8 h after co-transfection of let-7e mimic miRNA together with CASP3 reporter into HeLa cells significant (P<0.01, paired t test) and 35% decreased normalised luciferase activity (Luc *_Gaussia_* : Luc *_Cypridina_*) was measured compared to the non-sense miRNA transfected controls (figure 7 B). Sustained down-regulation (25%) was also detected at 24 h post transfection. Accordingly, HeLa cells co-transfected with miR-29a mimic miRNA together with CASP7 reporter showed significant (P<0.01, paired t test) and 25% decreased normalised luciferase activity after 8 h compared to the non-sense miRNA transfected controls (figure 7 B). Sustained down-regulation (27%) was also detected at 24 h post transfection. This approach showed that key regulators of apoptosis CASP3 and 7 are under the control of let-7e and miR-29a, respectively underlining the functional relevance of our strategy.

## Discussion

MAH represents a very diverse group of mycobacteria. Even within the same strain phenotypic variation occurs and is reflected by the appearance of different colony morphotypes. Different MAH strains and even different morphotypes of the same strain exhibit deviating virulence [10]. MAH occurs ubiquitously and has been isolated not only from patients but also from water, soil, dust, pig and other life stock [32]. The route of infection so far is unknown. A relatively high degree of genetic variability has been reported [33] but no correlation relating genetic traits or habitat on the one side and virulence on the other side has so far been established. Furthermore, there is no experimental evidence that environmental strains are less pathogenic than clinical strains. In order to take account of the diversity of MAH strains and to obtain a more representative view of the macrophage response upon MAH infection, we included in our study three MAH strains isolated from different settings. Strains 104 and 10091/06 were isolated form an HIV patient and a child with lymphadenitis, respectively, while strain 2514 was isolated from water.

In general, we deduce from our microarray data that both MAH strains isolated from human patients (10091/06 and 104) behave similar to other virulent mycobacteria independently from the pathogenesis of disease they caused in patients. In this regard we speculate that the pathogenesis of MAH-associated-diseases is rather a consequence of the immune conditions of patients than genetic differences of MAH determining pathogenicity. To give an example, two important members of the signalling events of host cells encountering pathogens are signal transducer and activator of transcription 1-alpha/beta (STAT1) and IFNGR1, which were timely regulated according to the expression data presented here (figure 1B, figure 4). It has been shown that pathogenic *M. avium* suppress IFNG and Jak/STAT signalling by decreasing levels of IFNGRs [34], which consequently reduced STAT1 phosphorylation and activity. This provides further indications that both studied MAH strains possess virulent characteristics by orchestrating diminished IFNG signalling. Gehring and colleagues moreover reported that the 19-kDa lipoprotein of *M. tuberculosis* inhibits IFNG-induced expression of FCGR1A [35]. They proposed that *M. tuberculosis* inhibits IFNGR signalling in human macrophages through 19-kDa lipoprotein activation of TLR2. Our data agrees with these observations since TLR2 was up-regulated after infection of MAH strains 2.6-fold after 24 h (figure 1 B). TLRs are receptors recognising pathogen-associated molecular patterns (PAMPs). Successive stimulation typically induces NFκB and MAPK pathways resulting in the production of pro-inflammatory cytokines, such as IL1B and TNF, finally inducing a T-cell response. Viable mycobacteria or lipopolysaccharide (LPS) induce the maturation of DCs probably through TLR2- and TLR4-dependent signalling pathways [36]. The protective effect of TLR2 signalling in mycobacterial infections was commonly proposed. On the other hand, TLR4 deficient mice were shown to behave like the wildtype in a *M. avium* infection model [37]. The observed TLR2 up-regulation and thereby mediated pro-inflammatory response supports the proposed major importance of TLR2 response in *M. avium* infection. Concomitant down-regulation of TLR4 indicates a subordinate role of TLR4 associated inflammatory response to MAH.

Upon infection, macrophages are functionally polarised into M1 and M2. Bacterial pathogens trigger the transcription of a set of genes belonging to the M1 program known as “common host response”. Progressive M1 polarisation, however, leads to excessive production of pro-inflammatory cytokines and results in tissue damage and recruitment of defence cells [25]. Pathogenic but not non-pathogenic mycobacteria have evolved mechanisms to suppress those signalling cascades (MAPK, Jak/STAT), which result in cytokine-induced immune response. Our data reflect both the expression of genes involved in protective immunity as well as inhibitors of activated macrophages. For example, IL1B has a protective role in immunity to tuberculosis and leads to the production of NO [38], [39]. IL10 blocks the pro-inflammatory cytokine production and reduces MHCII expression, which is required for antigen presentation. It furthermore inhibits activated DCs and macrophages [38]. Interestingly, M2 macrophages (derived from direct tumor cell contact and IL4, IL10 and IL13 exposure) drive a Th2 response resulting in increased expression of anti-inflammatory cytokines and down-regulation of pro-inflammatory mediators. M2 macrophages are characterised by IL4 and IL10 production, TGFB expression, and their involvement in tissue remodelling via VEGF and MMP expression [40]. Therefore, our data reflected an M2-like expression profile, which induces a Th2 response in order to evade the immune response and counteract inflammation. In this regard the early secreted antigenic target protein-6 (ESAT-6) from *M. tuberculosis* was reported to directly interfere with M1 polarisation by e.g. inhibiting NFκB activation [25]. On the other hand, our data pointed out the up-regulation of genes such as TNF, IL1B and the chemokine ligand CXCL10 that represent an M1 macrophage polarisation. The mixed M1/M2 activation represented by our data may be based on an initial M1 polarisation following the “common host response”, which is successively manipulated into M2 by ESAT-6-like proteins of MAH. In this context, Gey Van Pittius and colleagues have reported that multiple duplicates of the ESAT-6 gene cluster were found in *M. tuberculosis* and were conserved also in *M. leprae*, *M. bovis* and *M. avium* [41].

For validation of our microarray experiments we performed qRT-PCRs by including an environmental MAH isolate (2514) as well as *E. coli* K12 serving as a control. In general, we did not observe pronounced MAH strain specific response of host macrophages. This underlines our hypothesis that pathogenesis of disease is likely to be determined by the host than the MAH strain and its origin of isolation. However, LTA4H constituted an exception. LTA4H catalyses the synthesis of the pro-inflammatory eicosanoide leukotriene B4 (LTB4) that is a potent chemoattractant of polymorphonuclear leukocytes. One could speculate whether the water isolate MAH 2514 causes a preterm LTB4 derived inflammatory response compared to both MAH strains isolated from human patients. Our microarray experiments showed early PLCG2 down-regulation. PLCG2 was reported to be activated after infection with attenuated *M. tuberculosis* strains regulating several functions of neutrophils such as generation of reactive oxygen intermediates (ROI) and induction of MAPK-signalling [42]. Koul et al. moreover assumed that virulent *M. tuberculosis* may inhibit PLCG2 tyrosine phosphorylation resulting in down-regulation of MAPK signalling pathways. Interestingly, our microarray data showed direct transcriptional down-regulation of PLCG2 by both MAH strains probably leading to a similar outcome as hypothesised above. But consecutive qRT-PCRs (figure 4 A) rejected this hypothesis, since down-regulation of PLCG2 was also observed in the control MDMs infected with the non-pathogenic *E. coli* K12. Early transcriptional down-regulation of PLCG2 in host cells may constitute a general negative feedback loop avoiding excessive release of pro-inflammatory cytokines. In contrast to lacking MAH strain specific differences we observed donor specific variations of host response. These inter-individual differences call the use of cell lines such as THP-1, U937 or RAW 264.7 presented in several studies into question. Suchlike immortalised macrophage-like cell lines are altered especially with respect to cell survival. In our opinion, the use of primary macrophages derived from peripheral blood monocytes generates a more representative model of the host. Based on the observation of donor-specific differences, we suggest the randomisation of infection experiments using MDMs from different donors as presented in our study.

This is the first study investigating the macrophage miRNA-response to mycobacterial infections. Our study showed induction of renowned regulators of innate immunity miR-155 and miR-146 that modulate e.g. TLR signalling or cytokine response. As mentioned above, miR-146 targets TLR signalling molecules like TRAF6 [18], which is involved in TLR4 mediated inflammatory response. This is in consistence with our mRNA microarray data showing decreased TRAF6 levels in progressing infection (data not shown). As described in the introduction, these miRNAs are thought to direct a sharp macrophage immune response to antagonise pathogens but avoiding tissue damage by a negative feedback loop. However, three miRNAs (let-7e, miR-29a and miR-886-5p) were specifically up-regulated after mycobacterial infection at 48 h compared to 24 h. The here introduced approach of miRNA-mRNA-analysis is based on integration of expression data and target analysis and provides a theoretical concept for unveiling a new regulative layer, how distinct mycobacteria could alter host cell response to infection. To give an example, miR-886-5p was significantly up-regulated only at 48 h compared to *E. coli* K12 stimulation and we determined a potential interaction of miR-886-5p and IRAK3. Interestingly, the *M. tuberculosis* Man-LAM was reported to induce IRAK3 expression in macrophages by inhibiting IRAK-TRAF6 interaction [43]. While miR-886-5p expression was increased at 48 h, the IRAK3 expression was negatively correlated. Man-LAMs of distinct mycobacteria may cause differed IRAK3 response. It will be of interest, whether the mycobacterial mediated alteration of IRAK3 response is under the control of miR-886-5p. Our future study will concentrate on the question whether Man-LAMs of MAC are able to cause altered IRAK3 response of macrophages. The miR-29 family was shown to be transcriptionally repressed by hedgehog, NFκB and c-Myc [44]. Interestingly, the protein ESAT-6 from *M. tuberculosis* was shown to down-regulate the expression of c-Myc in an ERK1/2-dependent manner [45] and as mentioned above to drive an M2 polarisation. It would be interesting to address the question if mycobacteria are able to modulate host inflammatory response via the c-Myc mediated miR-29 pathway. MiR-29a was shown to directly target negative regulators of Wnt signalling [46]. On the other hand, Wnt is known to trigger macrophage inflammatory response [47]. As indicated above, we hypothesise an ESAT-6 dependent c-Myc-miR-29 axis, which may determine macrophage inflammatory response through modulation of Wnt signalling upon mycobacterial infection. Our network analysis showed mutual targeting of PDGFB by let-7e and miR-29a. PDGFB was shown to be one of the key molecules enabling the entry of an intracellular pathogen *Chlamydia pneumoniae* into human cells [48]. A PDGFB mediated uptake of mycobacteria has not been reported before; however, miR-29a/let-7e mediated down-regulation of PDGFB may be part of a protective mechanism of the host cell. Moreover, it was reported that CD44 enables binding of *M. tuberculosis* and mediates mycobacterial phagocytosis, macrophage recruitment and promotes protective immunity [49]. It will be to clarify whether pathogenic mycobacteria like *M. tuberculosis* are able to alter the CD44 induced processes via altering the let-7e expression.

Recently, the let-7 family as well as miR-886-5p were shown to regulate apoptosis of cancer cells by targeting BCL2L1 and BAX, respectively [50], [51]. As introduced, it was also shown that let-7a targets the executioner of apoptosis CASP3 [17]. Both the extrinsic and the intrinsic pathway of apoptotic cell death involve the activation of caspase cascade and degradation of genomic DNA. Apoptosis is controlled on many levels, and this involves the BCL2 family proteins, consisting of members with pro- and anti-apoptotic character. These regulate the release of cytochrome C (intrinsic pathway).Virulent strains of *M. tuberculosis* for example induce much lower levels of apoptosis than attenuated strains [52]. Apoptosis is an evolutionary old strategy of metazoans to eliminate pathogens by the loss of the infected cells to the benefit of the organism. It is assumed that virulent mycobacteria are able to inhibit apoptosis e.g. by altering the BCL2 pathway [53] as a possible mechanism to avoid host defence. On the other hand, induction of apoptosis in *M. avium*-infected macrophages limits bacterial viability based on the assumption that apoptosis acts as host defence mechanism [54]. However, the published data concerning the fate of infected cells is inconsistent. It was reported that intracellularly replicating *M. avium* strains induce apoptosis but other strains that are unable to multiply in macrophages cause apoptosis to a minor degree [55]. Many studies showed that induction of apoptosis by *M. avium* involves the extrinsic pathway and is TNF and TNFSF6 dependent. But other pathways involving ROI, p38 MAPK, apoptosis signal-regulating kinase I as well as disruption of the mitochondria seem to play a role in *M. avium*-induced apoptosis [56]. Programmed cell death in U937 cells infected with *M. avium* strain 101 was induced by 14-fold reduced expression of the anti-apoptopic gene BCL2L1 [14]. Our qRT-PCR data point out the ability of studied MAH strains to inhibit apoptosis by transcriptional down-regulation of pro-apoptotic members of the BCL2 family. In this context, the network deduced from both miRNA- as well as mRNA-qRT-PCR data revealed CASP3 and 7 to be targeted by let-7e and miR-29a, respectively. As demonstrated by the presented CASP3/CASP7 assays, both caspases possessed decreased activity after 6 h and 24 h p.i. in all samples compared to the non-infected control. However, sustained and significant diminished caspase activity was only caused by MAH strains at 48 h p.i. In line with this observation both miRNAs were up-regulated at 48 h p.i. and we were able to show that let-7e and miR-29a target both caspases. It seems that efficient and sustained inhibition of host macrophage apoptosis by mycobacteria relies on interplay of concomitant up-regulation of let-7e and miR-29a targeting key caspases along with increased expression of miR-886-5p mediating the down-regulation of the apoptotic activator BAX [50].

Taken together, we show for the first time that miRNAs are specifically induced after mycobacterial infection of human macrophages compared to *E. coli* K12 stimulation. We provide a theoretical concept of gene expression regulation in the host response to mycobacteria and functional relevance of presented data is exemplified by the let-7e/miR-29a mediated down-regulation of CASP3 and CASP7. We furthermore point out that MAH-induced up-regulation of miRNA expression interferes with regulation of apoptosis. Since various intracellular bacterial pathogens are able to manipulate host apoptotic pathways differently, our ongoing work will address the question if different agents cause a specific miRNA response.

## Materials and Methods

### Bacterial strains and cultivation

MAH strain 104 is an isolate from an AIDS patient [21]. MAH strain 10091/06 was isolated from the lymph node of an infected child. Both strains were kindly provided by Dr. Elvira Richter from the National Reference Center for mycobacteria in Borstel, Germany. MAH strain 2514 was isolated from drinking water and was kindly provided by Prof. Schulze-Röbbecke, University Hospital Düsseldorf, Germany. *E. coli* K12 as well as mycobacteria were grown as described before [57].

### Infection of human monocyte derived macrophages

Monocytes were isolated from buffy coats from healthy donors using Ficoll-Paque™ Plus (GE Healthcare) and Percoll™ (GE Healthcare) gradient centrifugation according to the manufacturer's recommendations. After the Percoll gradient centrifugation, the monocytes were washed twice with PBS (140 mM NaCl, 16.0 mM Na_2_HPO_4_, 2.00 mM KH_2_PO_4_, 3.75 mM KCl, pH 7.4) and resuspended in IMDM medium (PAA) with 3% human AB serum. The cells were counted and 6 to 10 million cells were seeded per 25 cm^2^ cell culture flask (TPP) and incubated at 37°C and in 5% CO_2_. Cells were allowed to adhere overnight and the non-adherent cells were then removed by sound rinsing of the cell layer using IMDM medium.

To quantify the proportion of monocytes as well as MDMs in the adherent cell fraction, the cells in one of the flasks were detached by addition of 20 ml PBS with 5 mM EDTA, incubation at 4°C for 1 to 2 h and scraping off. After centrifugation the cells were resuspended in PBS and the percentage of monocytes was determined by FACS analysis using a mouse anti-human CD14 antibody (monoclonal antibody MEM-18, Immuno Tools) and a goat anti-mouse FITC-conjugated secondary antibody (ImmunoTools). A mouse IgG1 control (monoclonal antibody 203, Immuno Tools) was included to assess non-specific antibody binding. FACS analysis was performed using the BD FACScalibur cytometer (BD Biosciences).

The cells in the remaining flasks were allowed to differentiate during three days in IMDM with 3% AB serum and then infected for 4 h with MAH 104, MAH 10091/06, MAH 2514 or *E. coli* K12 DH5α at a multiplicity of infection (MOI) of 25 as described earlier with following modifications [58]. One flask was not inoculated and served as negative control. Non-phagocytosed bacteria were removed by washing and treating the cell layer with 200 µg/ml amikacin for 2 h and final incubation in medium containing 5 µg/ml amikacin. Samples were taken for RNA isolation after 6 h, 24 h and 48 h p.i. All strains were used to perform three independent infection experiments applying MDMs from different human donors. Total RNA was isolated from infected as well as non-infected adherent cells using the miRVana miRNA Isolation Kit (Life Technologies) as described earlier [59]. The described procedure was performed in triplicate using the buffy coats from three independent healthy donors.

Apoptosis of monocytes was monitored by measurement of activity of caspases 3 and 7 using the Caspase-Glo® 3/7 Assay System (Promega) according to the manufacturer's protocol.

### RNA amplification

In order to generate sufficient amounts of RNA for cDNA microarray experiments, pools of RNA (from triplicate infection experiments) were amplified using the MessageAmp II aRNA Amplification Kit (Life Technologies) according to the manufacturer's instructions. To compensate the donor based biological variance, pools of total RNA from three independent infection experiments were prepared for MAH strains 104 and 10091/06 and non-infected controls relating to each point in time (6, 24 and 48 h p.i.). Each infection experiment was performed with primary monocytes derived from the buffy coats of three different human donors. The procedure included a reverse transcription using 1 µg of pooled total RNA and a T7 oligo (dT) primer to synthesise first strand cDNA containing a T7 promoter sequence. The cDNA is subsequently converted into double stranded DNA (dsDNA) by second strand synthesis. After degradation of RNA and cDNA purification, in vitro transcription (IVT) was employed to generate antisense amplified RNA (aRNA). After a final purification step quality and quantity of aRNA samples were assessed using Agilent 2100 Bioanalyzer and RNA Nano Chips (Agilent) and Nanodrop 1000 Spectrophotometer (Thermo Scientific).

### cDNA microarray analysis

Experiments were performed using the PIQOR™ Immunology Microarray Kit, human, antisense (Miltenyi Biotec). The microarray carries 1076 cDNA probes, which are 200–400 bp in length and spotted in quadruplicate. PIQOR™ Immunology Microarrays include key genes for immune response, cell death, extracellular matrix, and signal transduction as well as 6 housekeeping genes and 6 controls. All hybridisations were accomplished using the generated aRNA samples as two colour experiments hybridising two samples labelled with different fluorescent dyes to a single microarray. aRNA was converted into cDNA and fluorescently labelled using the SuperScript™ Plus Indirect cDNA Labelling System (Life Technologies) and Alexa Fluor® Dyes. 15 µg aRNA and anchored oligo (dT) were used for reverse transcription according to the manufacturer's protocol. Sample cDNA (infected) was fluorescently labelled with Alexa Fluor® 647 (red emission), while the control (non-infected) was labelled with Alexa Fluor® 555 (green emission). Dye-dependent bias was evaluated by a dye swap experiment. Automated hybridisation and washings were carried out in the aHyb™ Hybridisation Station (Miltenyi Biotec). Buffers for pre-hybridisation, hybridisation and wash were supplied with the PIQOR™ Microarray Kit (Miltenyi Biotec) and protocols were followed according to the manufacturer's instructions. Briefly, initial pre-treatment was performed at 75°C for 2 min using 50% formamide in bi-distilled water followed by 2 wash cycles with bi-distilled water at 75°C for 2 min; pre-hybridisation was performed at 63°C for 5 min and fluorescently labelled samples were hybridised for 16 hours at 63°C. Finally the slides were successively washed for 1 minute (2 cycles) with the supplied wash buffer 1 and 2 at 50°C and 35°C, respectively. Afterwards, slides were removed from the hybridisation station and dipped several times in bi-distilled water. Finally, the slides were dried by brief centrifugation. The microarrays were scanned at 10 µm resolution using a GenePix 4000B scanner (Molecular Devices). Microarray image analysis and ratio-based normalisation of the microarray data was conducted using the software package GenePix Pro 6.1 (Molecular Devices). In order to avoid analysis of weakly and unexpressed transcripts, criteria were defined to select genes for further analysis based on background-subtracted signal intensity, signal to noise ratio and spot diameter. After assessment of reproducibility and evaluation of dye swap experiments replicate microarray data was combined by calculating the mean of respective log 2 ratios for subsequent analysis. Data analysis was performed using the Acuity 4.0 software package (Molecular Devices). Discrimination of expression profiles reflecting temporal regulation during the course of infection was assessed by statistical analysis of different time points after infection using ANOVA. The data was organised using cluster analysis and displayed as individual heatmaps according to median log 2 ratios of all time points.

MIAME-compliant data of all performed microarrays considering the applied platforms as well as processed and raw sample data were submitted to the NCBI GEO repository [60] and accession-numbers were assigned (series: GSE27100; cDNA microarrays: GSM671371-76).

### Quantification of mRNA

Quantification of gene expression was performed by means of qRT-PCR as described earlier with few modifications [61]. Firstly, 1 µg of total RNA was reverse-transcribed using the RevertAid™ M-MuLV Reverse Transcriptase (Fermentas GmbH) in 20 µl total volume using random Hexamers. SYBR Green qPCR was performed using the SensiMix DNA Kit (Quantace Ltd.) and 0.2 µM of gene specific primers (table S3). The first step of amplification was denaturation at 95°C for 10 min, followed by 40 cycles with 15 s at 95°C, 10 s at 60°C and 20 s at 72°C. The fluorescence signal was acquired at 72°C. Melting curve analysis allowed testing for specificity of qRT-PCR. The optimal annealing temperatures of all primers (table S3) were first evaluated performing gradient qRT-PCRs followed by sequencing of purified amplicons. Absolute quantification was performed by converting the Ct values into fg of the specific amplicon per qRT-PCR reaction using a calibration curve, which was established by serial dilutions of the corresponding PCR product. Gene expression was quantified by triplicate measurements of 1 µl 1∶5 diluted cDNA in 10 µl final reaction volume. All reactions were run in the StepOnePlus™ Real-Time PCR System (Life Technologies). Normalisation of expression data was performed using the geNorm algorithm [62] that is based on calculation of the geometric means of several housekeeping genes (in this study we used 18S rRNA, GAPDH, and ACTB all possessing stable expression). The relative gene expression was obtained by calculating the ratios of absolute quantification values and normalisation factors provided by geNorm. Subsequent heatmap analysis was performed using the MultiExperiment Viewer (MeV) from the TM4 software package [63]. For this purpose, the log 2 ratio of means (triplicate qRT-PCR measurements) of infected samples and non-infected controls were calculated and were subjected to the MeV analysis. Independent heatmaps were created for every point of sampling (6, 24 and 48 h p.i.).

### miRNA microarray analysis

MiRNA expression in infected human monocytes was discovered using the miRCURY LNA™ miRNA Array kit v. 10.0 (Exiqon) with the miRCURY LNA™ miRNA Array Power Labeling kit (Exiqon). One microarray consists of eight sub-arrays composed of tm-normalised capture probes complementary to mature miRNAs in four replicates. In order to balance possible biological variation, hybridisations were performed using pooled total RNA samples. For this purpose, pools of total RNA from three independent infection experiments were prepared for both MAH strains (104 and 10091/06) and non-infected controls relating to each point in time (6, 24 and 48 h p.i.). Each infection experiment was performed with MDMs of individual human donors. All microarray experiments were realised as two colour experiments hybridising two samples labelled with different fluorescent dyes to a single array. Our general internal laboratory convention (except of dye swap experiments) implied that samples (RNA from infected cells) were labelled always with Hy5, while the control (RNA from non-infected cells) was labelled with Hy3. Fully automated hybridisation and washings were carried out in the aHyb™ Hybridsation Station (Miltenyi Biotec). Buffers for pre-hybridisation, hybridisation and washes were supplied with the miRCURY LNA™ miRNA Array kit. Slides were first pre-hybridised at 56°C for 5 min and fluorescently labelled samples were hybridised for 16 hours at 56°C. Afterwards, the slides were washed twice for 1 minute with the supplied wash buffer A at 56°C followed by two successive washes at 23°C using wash buffers B and C, respectively. Afterwards, slides were removed from the hybridisation station and dipped in bi-distilled water and dried by brief centrifugation. Image acquisition and data analysis was performed as described above.

MIAME-compliant data of all performed microarrays considering the applied platforms as well as processed and raw sample data were submitted to the NCBI GEO repository [60] and accession-numbers were assigned (series: GSE27100; miRNA microarrays: GSM669466-71).

### Quantification of miRNA

The quantification of miRNA expression in individual samples was carried out by means of a miRNA specific qRT-PCR approach called miR-Q as described earlier [30] using the oligonucleotides in table S3. MiRNA quantification was performed by triplicate measurements for each sample, compared with a calibration curve established by reverse transcription of serially diluted amounts of the particular synthetic miRNA in the presence of 50 ng bacterial total RNA. The RT reaction of non-spiked bacterial total RNA samples and no template controls were used as negative controls. All reactions were run in the StepOnePlus™ Real-Time PCR System (Life Technologies). Normalisation of expression data was performed using the geNorm algorithm as described above. The geometric means of three miRNAs (miR-16, miR-21 and miR-24), which showed stable expression on miRNA microarrays was used for calculation of the normalisation factor. The relative gene expression was obtained by calculating the ratios of absolute quantification values and normalisation factors provided by geNorm.

### Transfection and reporter gene assays

The human cervix carcinoma cell line HeLa (ATCC No. CCL-2) was maintained in RPMI 1640 (Biochrom AG) supplemented with 10% fetal bovine serum superior (Biochrom AG) and 10 µg/ml Gentamicin (Biochrom AG) and passaged twice weekly. Cultivation of cells was performed in 75 cm^2^ flasks (Greiner Bio-One GmbH) at 37°C and 5% CO_2_. HeLa cells were transfected using the Nucleofector Technology (Lonza AG). Nucleofection was performed using 5×10^5^–1×10^6^ HeLa using 0.9–1.8 µg reporter plasmid (pTK-Gluc derivatives, NEB GmbH), 100–200 ng normalisation plasmid (pTK-Cluc, NEB GmbH) and 100 pmol miRNA mimic according to the manufacturer's instructions.

For generation of reporter plasmids, the 3′ UTRs of human CASP3 and 7 were amplified using the oligonucleotides NotIhCASP3-3UTRf, XhoIhCASP3-3UTRr, NotIhCASP7-3UTRf and XhoIhCASP7-3UTRr (table S3). The amplicons were cloned in pTK-Gluc (NEB GmbH) using the restriction enzymes NotI and XhoI (NEB GmbH). 100 ng of linearised pTK-Gluc and 50 ng of digested 3′ UTR were ligated in 20 µl total volume using T4 DNA Ligase (NEB GmbH) for 30 min at 22°C followed by heat inactivation at 65°C for 10 min. *E. coli* K12 were transformed and positive clones harbouring the 3′ UTRs of CASP3 and CASP7 were selected and sequenced, respectively. Endotoxin-free reporter plasmids (pTKGhCASP3 and pTKGhCASP7) were produced for transfection using NucleoBond Xtra Midi Plus EF (Macherey-Nagel GmbH & Co. KG). The reporter plasmids pTKGhCASP3 or pTKGhCASP7 were cotransfected with Pre-miR miRNA Precursors hsa-let-7e or hsa-miR-29a (Life Technologies), respectively. The non-sense miRNA Pre-miR miRNA Precursor Negative Control #1 (Life technologies) was used as a control for specificity of interaction. Detection of Gaussia and Cypridina Luciferase activity serving as experimental and control reporter genes was performed using the Biolux Assay Kits (NEB GmbH). Supernatants of adherent cells were taken at 8 and 24 h post Nucleofection. Luciferase measurement of three independent transfection experiments was performed in triplicate using 15 µl of the supernatant according to the manufacturer's protocol. Luciferase activity was determined in white 96 well microplates (Greiner Bio-One GmbH) using the automated luminometer FLUOstar OPTIMA (BMG Labtech).

## Supporting Information

Table S1 **Pathways affected by predicted targets of expressed miRNAs.**
(DOC)

Table S2 **Predicted interactions of negatively correlating miRNA and mRNAs.**
(DOC)

Table S3 **Oligonucleotides used in this work.**
(XLS)
