# Supplementary material for: Integrated MicroRNA-mRNA-Analysis of Human Monocyte Derived Macrophages upon Mycobacterium avium subsp. hominissuis Infection
Source: PLoS One. 2011 May 24;6(5):e20258. doi: 10.1371/journal.pone.0020258 (PMC3101234; doi:10.1371/journal.pone.0020258)
Supplement: Table S1 — Pathways affected by predicted targets of expressed miRNAs. (DOC) [file pone.0020258.s001.doc]

Supplementary table 1: Pathways affected by predicted targets of expressed microRNAs.

| **Pathways potentially affected by predicted target genes*** | **Predicted target genes**** | **% of genes***** |
| --- | --- | --- |
| CCR3 signaling in Eosinophils | MAPK1, PLCB1, PPP1R12B, ROCK2 | 18 |
| PKC-catalyzed phosphorylation of inhibitory phosphoprotein of myosin phosphatase | ARHGAP5, GNA13, PLCB1, PPP1R12B | 31 |
| Rho cell motility signaling pathway | AICDA, ARHGAP5, BAIAP2, PPP1R12B | 17 |
| Human Cytomegalovirus and Map Kinase Pathways | MAP3K1, MAPK1, MAPK14, RB1, SP1 | 36 |
| Mechanism of Gene Regulation by Peroxisome Proliferators via PPARa(alpha) | DUSP1, NCOR2, NRIP1, PPARA, PPARGC1A, RB1, SP1 | 17 |
| CARM1 and Regulation of the Estrogen Receptor | CCND1, ESR1, GTF2A1, NCOR2, NRIP1, PPARGC1A, TBP | 28 |
| Chromatin Remodeling by hSWI/SNF ATP-dependent Complexes | GTF2A1, NF1, SMARCC1, TBP | 24 |
| Nuclear receptors coordinate the activities of chromatin remodeling complexes and coactivators to facilitate initiation of transcription in carcinoma cells | GTF2A1, KAT2B, NCOA2, NCOA3, NCOR2, TBP | 40 |
| Control of Gene Expression by Vitamin D Receptor | CHAF1A, KAT2B, NCOA2, NCOA3, SMARCC1 | 19 |
| Regulation of BAD phosphorylation | BCL2, BCL2L1, IGF1, IGF1R, IL3, MAPK1 | 33 |
| Role of Mitochondria in Apoptotic Signaling | APAF1, BCL2, BCL2L1, CASP3, CASP7 | 24 |
| Telomeres, Telomerase, Cellular Aging, and Immortality | BCL2, IGF1R, KRAS, RB1, TNKS, TP53 | 33 |
| Influence of Ras and Rho proteins on G1 to S Transition | CCND1, CCNE1, CDK6, E2F1, MAPK1, RB1 | 25 |
| Apoptotic Signaling in Response to DNA Damage | APAF1, BCL2, BCL2L1, CASP3, CASP7, PXDN, TP53 | 33 |
| Induction of apoptosis through DR3 and DR4/5 Death Receptors | APAF1, BCL2, CASP3, CASP7, MAP3K14 | 18 |
| FAS signaling pathway ( CD95 ) | CASP3, CASP7, FAS, MAP2K4, MAP3K1, PKN2, RB1 | 23 |
| Stress Induction of HSP Regulation | APAF1, BCL2, CASP3, FAS, MAPKAPK3 | 36 |
| p53 Signaling Pathway | APAF1, BCL2, CCND1, CCNE1, E2F1, RB1, TP53 | 44 |
| Regulation of transcriptional activity by PML | FAS, RB1, SIRT1, TP53 | 27 |
| Estrogen-responsive protein Efp controls cell cycle and breast tumors growth | CDK6, ESR1, SMURF1, TP53 | 57 |
| Tumor Suppressor Arf Inhibits Ribosomal Biogenesis | ABL1, E2F1, RB1, TP53 | 33 |
| BTG family proteins and cell cycle regulation | BTG2, CCND1, NGF, RB1, TP53 | 50 |
| Cyclins and Cell Cycle Regulation | CCND1, CCND2, CCNE1, CDK6, E2F1, RB1, RBL1 | 30 |
| METS affect on Macrophage Differentiation | E2F1, ETS1, FOS, NCOR2, RBL1 | 31 |
| Cyclin E Destruction Pathway | CCNE1, CDC34, E2F1, FBXW7, RB1 | 56 |
| Cell Cycle: G1/S Check Point | ABL1, CCND1, CCNE1, CDK6, E2F1, RB1, SMAD4, TP53 | 31 |
| E2F1 Destruction Pathway | CCNE1, CDC34, E2F1, RB1 | 40 |
| ALK in cardiac myocytes | APC, BMP2, SMAD1, SMAD4, SMAD5, TGFBR1, TGFBR2, TGFBR3, WNT1 | 26 |
| NFkB activation by Nontypeable Hemophilus influenzae | DUSP1, IKBKB, MAP3K14, MAPK14, SMAD4, TGFBR1, TGFBR2 | 29 |
| TGF beta signaling pathway | APC, SMAD2, SMAD4, TGFBR1, TGFBR2, ZFYVE9 | 35 |
| CTCF: First Multivalent Nuclear Factor | CTCF, PTEN, SMAD1, SMAD4, SMAD5, TGFBR3, TP53 | 39 |
| Inactivation of Gsk3 by AKT causes accumulation of b-catenin in Alveolar Macrophages | APC, CCND1, IRAK1, LEF1, PDPK1, WNT1 | 23 |
| WNT Signaling Pathway | APC, CCND1, NLK, PPARD, SMAD4, WNT1 | 24 |
| Ceramide Signaling Pathway | BCL2, MAP2K4, MAP3K1, MAPK1 | 19 |
| Control of skeletal myogenesis by HDAC & calcium/calmodulin-dependent kinase (CaMK) | IGF1, IGF1R, MAPK14, NFATC1 | 24 |
| Skeletal muscle hypertrophy is regulated via AKT/mTOR pathway | IGF1, IGF1R, PDPK1, PTEN | 20 |
| Multiple antiapoptotic pathways from IGF-1R signaling lead to BAD phosphorylation | GRB2, IGF1R, IRS1, MAPK1, SOS1 | 28 |
| The IGF-1 Receptor and Longevity | FOXO3, GHR, IGF1, IGF1R | 36 |
| Erythrocyte Differentiation Pathway | CCL3, FLT3, IGF1, IL3 | 31 |
| Roles of ÃŸ-arrestin-dependent Recruitment of Src Kinases in GPCR Signaling | ADRBK1, KCNA1, MAPK1, PLCB1 | 22 |
| Role of ÃŸ-arrestins in the activation and targeting of MAP kinases | ADRBK1, KCNA1, MAPK1, PLCB1 | 29 |
| Phospholipids as signalling intermediaries | ITGAV, ITGB3, MAPK1, PDGFRA, PLCB1 | 22 |
| fMLP induced chemokine gene expression in HMC-1 cells | MAP3K1, MAPK1, MAPK14, NFATC1, PLCB1 | 20 |
| Integrin Signaling Pathway | ACTN2, CRKL, GRB2, MAPK1, PPP1R12B, PXDN, SOS1, TNS1 | 24 |
| ÃŸ-arrestins in GPCR Desensitization | ADRBK1, KCNA1, PLCB1, PPARA | 36 |
| The 4-1BB-dependent immune response | IKBKB, MAP3K1, MAPK14, TNFSF9 | 24 |
| Angiotensin II mediated activation of JNK Pathway via Pyk2 dependent signaling | GRB2, MAP2K4, MAP3K1, MAPK1, SOS1 | 19 |
| HIV-I Nef: negative effector of Fas and TNF | APAF1, BCL2, CASP2, CASP3, CASP7, FAS, MAP3K1, MAP3K14, PKN2, RB1, SYNGAP1 | 22 |
| TPO Signaling Pathway | FOS, GRB2, SOS1, STAT3, SYNGAP1 | 25 |
| AKT Signaling Pathway | FOXO1, FOXO3, GHR, PDPK1 | 21 |
| Oxidative Stress Induced Gene Expression Via Nrf2 | FOS, MAFG, MAFK, MAPK1, MAPK14 | 28 |
| B Cell Survival Pathway | CASP3, CASP7, DPF2, FOS | 31 |
| BCR Signaling Pathway | FOS, GRB2, MAP3K1, MAPK14, NFATC1, SOS1 | 23 |
| Bioactive Peptide Induced Signaling Pathway | GRB2, MAPK1, MAPK14, MAPT, SOS1 | 17 |
| Caspase Cascade in Apoptosis | APAF1, CASP2, CASP3, CASP7 | 17 |
| CD40L Signaling Pathway | DUSP1, IKBKB, MAP3K1, MAP3K14, TNFAIP3, TRAF6 | 43 |
| Transcription factor CREB and its extracellular signals | GRB2, MAPK1, MAPK14, SOS1 | 22 |
| EGF Signaling Pathway | FOS, GRB2, JAK1, MAP2K4, MAP3K1, SOS1, STAT3, SYNGAP1 | 31 |
| Regulation of eIF4e and p70 S6 Kinase | GHR, IRS1, MAPK1, MAPK14, MKNK1, PDPK1, PTEN | 35 |
| Erk1/Erk2 Mapk Signaling pathway | GRB2, IGF1R, MAPK1, MKNK1, MKNK2, NGF, PDGFRA, SOS1, STAT3 | 32 |
| Fc Epsilon Receptor I Signaling in Mast Cells | FOS, GRB2, MAP2K4, MAP3K1, MAPK1, NFATC1, PKN2, SOS1 | 27 |
| Growth Hormone Signaling Pathway | GHR, GRB2, IRS1, SOS1 | 18 |
| Inhibition of Cellular Proliferation by Gleevec | CRKL, FOS, GRB2, MAP2K4, MAP3K1, SOS1 | 30 |
| Role of ERBB2 in Signal Transduction and Oncology | ERBB4, ESR1, GRB2, IL6R, MAPK1, SOS1, STAT3 | 33 |
| IGF-1 Signaling Pathway | FOS, GRB2, IGF1, IGF1R, IRS1, SOS1, SYNGAP1 | 37 |
| Signal transduction through IL1R | IKBKB, IL1RAP, MAP3K1, MAP3K14, MAPK14, TRAF6 | 21 |
| IL 2 signaling pathway | FOS, GRB2, JAK1, SOS1 | 19 |
| IL-2 Receptor Beta Chain in T cell Activation | BCL2, BCL2L1, CBL, CRKL, E2F1, FAS, FOS, GRB2, IRS1, JAK1, SOS1 | 32 |
| IL 3 signaling pathway | FOS, GRB2, IL3, SOS1 | 29 |
| IL 6 signaling pathway | FOS, GRB2, IL6R, SOS1, STAT3 | 26 |
| Insulin Signaling Pathway | FOS, GRB2, IRS1, SOS1, SYNGAP1 | 26 |
| Keratinocyte Differentiation | BCL2, ETS1, FAS, IKBKB, MAP2K4, MAP3K1, MAP3K14, MAPK1, MAPK14, SP1 | 27 |
| mTOR Signaling Pathway | EIF4B, FKBP1A, MKNK1, PDPK1, PTEN, TSC1 | 29 |
| MAPKinase Signaling Pathway | FOS, GRB2, IKBKB, MAP2K4, MAP3K1, MAP3K14, MAP3K2, MAP3K3, MAP3K9, MAP4K3, MAPK1, MAPK14, MAPK4, MAPK6, MAPKAPK3, MAX, MKNK1, MKNK2, PKN2, RAPGEF2, RPS6KA2, RPS6KA3, SP1, TGFBR1 | 30 |
| Signaling of Hepatocyte Growth Factor Receptor | CRKL, GAB1, GRB2, MAPK1, PTEN, PXDN, RAP1B, SOS1, STAT3, SYNGAP1 | 31 |
| NFAT and Hypertrophy of the heart (Transcription in the broken heart) | EDN1, FKBP1A, HAND1, IGF1, LIF, MAPK1, MAPK14, NFATC1 | 22 |
| NF-kB Signaling Pathway | IKBKB, IRAK1, MAP3K1, MAP3K14, TNFAIP3, TRAF6 | 27 |
| Nerve growth factor pathway (NGF) | FOS, GRB2, NGF, SOS1 | 25 |
| p38 MAPK Signaling Pathway | CDC42, GRB2, MAP2K4, MAP3K1, MAP3K9, MAPK14, MAX, MKNK1, RAPGEF2, TGFBR1 | 29 |
| PDGF Signaling Pathway | FOS, GRB2, JAK1, MAP2K4, MAP3K1, PDGFRA, SOS1, STAT3, SYNGAP1 | 36 |
| PTEN dependent cell cycle arrest and apoptosis | FOXO3, GRB2, MAPK1, PDPK1, PTEN, SOS1 | 33 |
| Links between Pyk2 and Map Kinases | CRKL, GRB2, MAP2K4, MAP3K1, MAPK1, MAPK14, SOS1 | 28 |
| Sprouty regulation of tyrosine kinase signals | CBL, GRB2, MAPK1, SOS1, SYNGAP1 | 33 |
| TNF/Stress Related Signaling | CASP2, IKBKB, MAP2K4, MAP3K1, MAP3K14, MAPK14 | 25 |
| T Cell Receptor Signaling Pathway | FOS, GRB2, MAP2K4, MAP3K1, NFATC1, SOS1, SYNGAP1 | 20 |
| Trefoil Factors Initiate Mucosal Healing | APAF1, GHR, GRB2, MAPK1, SOS1 | 23 |
| TNFR1 Signaling Pathway | CASP2, CASP3, MAP2K4, MAP3K1, PKN2, RB1 | 21 |
| TNFR2 Signaling Pathway | DUSP1, IKBKB, MAP3K1, MAP3K14, TNFAIP3, TNFRSF1B | 35 |
| Toll-Like Receptor Pathway | FOS, IKBKB, IRAK1, MAP2K4, MAP3K1, MAP3K14, MAP3K7IP2, MAPK14, PPARA, TRAF6 | 28 |
| TSP-1 Induced Apoptosis in Microvascular Endothelial Cell | CASP3, FOS, MAPK14, THBS1 | 57 |
| Role of Tob in T-cell activation | CD28, SMAD4, TGFBR3, TOB1 | 27 |
| Synaptic Proteins at the Synaptic Junction | ADD1, ANK3, EPB41, MPP2, NFASC | 29 |
| Agrin in Postsynaptic Differentiation | AGRN, ARHGEF6, CDC42, DMD, MAPK1, PXDN, SP1, UTRN | 30 |
| ADP-Ribosylation Factor | ARAP2, ARFGEF1, ASAP1, CYTH3 | 24 |
| Role of PI3K subunit p85 in regulation of Actin Organization and Cell Migration | AICDA, CDC42, PDGFRA, WASL | 44 |
| Dicer Pathway | DICER1, EIF2C1, EIF2C3, EIF2C4 | 80 |
| Selective expression of chemokine receptors during T-cell polarization | CCL3, CCR7, CD28, CNOT6 | 15 |
| Rac 1 cell motility signaling pathway | CDK5R1, MAP3K1, PDGFRA, PPP1R12B | 19 |

* Pathways containing at least 4 genes (or 8 %) predicted to be targeted by miRNAs showing valid expression at all time points and considering both MAH strains (10091/06 and 104).

** Predicted target genes with shared pathway memberships. Target Scan and PITA were used as algorithms for target prediction using the web tool MAGIA.

*** Percentage of the predicted target genes compared to all pathway-associated genes according to Reactome/Biocarta and Kegg pathway databases implemented in Cytoscape 2.7.0.
