# Supplementary material for: Integrated MicroRNA-mRNA-Analysis of Human Monocyte Derived Macrophages upon Mycobacterium avium subsp. hominissuis Infection
Source: PLoS One. 2011 May 24;6(5):e20258. doi: 10.1371/journal.pone.0020258 (PMC3101234; doi:10.1371/journal.pone.0020258)
Supplement: Table S2 — Predicted interactions of negatively correlating miRNA and mRNAs. (DOC) [file pone.0020258.s002.doc]

Supplementary table 2: Predicted interactions of negatively correlating miRNA and mRNAs.

| **Entrez Gene ID*** | **Gene Sympol*** | **miRNA**** | **Pearson r***** |
| --- | --- | --- | --- |
| 960 | CD44 | hsa-let-7e | -0.85 |
| 3586 | IL10 | hsa-let-7e | -0.83 |
| 3576 | IL8 | hsa-let-7i | -0.79 |
| 3586 | IL10 | hsa-let-7f | -0.77 |
| 3586 | IL10 | hsa-let-7g | -0.77 |
| 3576 | IL8 | hsa-mir-20a | -0.75 |
| 5597 | MAPK6 | hsa-let-7e | -0.71 |
| 5597 | MAPK6 | hsa-let-7i | -0.68 |
| 7130 | TNFAIP6 | hsa-mir-23a | -0.67 |
| 7531 | YWHAE | hsa-mir-886-5p | -0.66 |
| 6367 | CCL22 | hsa-mir-374a | -0.65 |
| 3576 | IL8 | hsa-let-7a | -0.64 |
| 6367 | CCL22 | hsa-let-7f | -0.64 |
| 7534 | YWHAZ | hsa-mir-22 | -0.6 |
| 1236 | CCR7 | hsa-let-7g | -0.6 |
| 3925 | STMN1 | hsa-mir-193a-3p | -0.59 |
| 3586 | IL10 | hsa-let-7i | -0.57 |
| 836 | CASP3 | hsa-let-7i | -0.57 |
| 5597 | MAPK6 | hsa-let-7f | -0.54 |
| 7130 | TNFAIP6 | hsa-mir-23b | -0.54 |
| 6367 | CCL22 | hsa-mir-21 | -0.53 |
| 4087 | SMAD2 | hsa-let-7e | -0.53 |
| 836 | CASP3 | hsa-let-7e | -0.53 |
| 4087 | SMAD2 | hsa-mir-16 | -0.51 |
| 3576 | IL8 | hsa-let-7g | -0.51 |
| 7531 | YWHAE | hsa-mir-29a | -0.5 |
| 6367 | CCL22 | hsa-let-7e | -0.49 |
| 4780 | NFE2L2 | hsa-mir-155 | -0.49 |
| 9435 | CHST2 | hsa-mir-886-3p | -0.48 |
| 960 | CD44 | hsa-mir-21 | -0.48 |
| 3576 | IL8 | hsa-let-7f | -0.46 |
| 1509 | CTSD | hsa-mir-185 | -0.45 |
| 4087 | SMAD2 | hsa-mir-21 | -0.45 |
| 3576 | IL8 | hsa-let-7e | -0.44 |
| 1236 | CCR7 | hsa-mir-21 | -0.44 |
| 3586 | IL10 | hsa-mir-27a | -0.44 |
| 7531 | YWHAE | hsa-mir-155 | -0.44 |
| 5597 | MAPK6 | hsa-let-7g | -0.43 |
| 6346 | CCL1 | hsa-mir-20a | -0.43 |
| 6367 | CCL22 | hsa-let-7i | -0.42 |
| 1236 | CCR7 | hsa-let-7f | -0.41 |
| 8741 | TNFSF13 | hsa-mir-185 | -0.41 |
| 1236 | CCR7 | hsa-let-7i | -0.41 |
| 4775 | NFATC3 | hsa-mir-185 | -0.39 |
| 1230 | CCR1 | hsa-mir-185 | -0.39 |
| 7534 | YWHAZ | hsa-let-7e | -0.38 |
| 836 | CASP3 | hsa-let-7f | -0.36 |
| 10068 | IL18BP | hsa-mir-185 | -0.36 |
| 8763 | CD164 | hsa-mir-16 | -0.34 |
| 6346 | CCL1 | hsa-mir-21 | -0.34 |
| 4087 | SMAD2 | hsa-mir-886-5p | -0.32 |
| 5597 | MAPK6 | hsa-let-7a | -0.32 |
| 4170 | MCL1 | hsa-mir-193a-3p | -0.31 |
| 5579 | PRKCB | hsa-mir-185 | -0.31 |
| 1236 | CCR7 | hsa-let-7e | -0.31 |
| 4087 | SMAD2 | hsa-let-7f | -0.31 |
| 836 | CASP3 | hsa-let-7g | -0.3 |
| 7531 | YWHAE | hsa-let-7e | -0.29 |
| 4170 | MCL1 | hsa-mir-29a | -0.28 |
| 7534 | YWHAZ | hsa-mir-193a-3p | -0.28 |
| 637 | BID | hsa-mir-26b | -0.27 |
| 1026 | CDKN1A | hsa-mir-423-5p | -0.27 |
| 8554 | PIAS1 | hsa-mir-886-5p | -0.27 |
| 5606 | MAP2K3 | hsa-mir-21 | -0.26 |
| 7534 | YWHAZ | hsa-let-7i | -0.25 |
| 4087 | SMAD2 | hsa-let-7i | -0.24 |
| 839 | CASP6 | hsa-mir-185 | -0.23 |
| 3604 | TNFRSF9 | hsa-mir-886-3p | -0.22 |
| 6367 | CCL22 | hsa-let-7a | -0.21 |
| 4087 | SMAD2 | hsa-let-7g | -0.21 |
| 5155 | PDGFB | hsa-mir-29a | -0.2 |
| 3678 | ITGA5 | hsa-mir-423-5p | -0.19 |
| 960 | CD44 | hsa-mir-423-5p | -0.18 |
| 1230 | CCR1 | hsa-mir-423-5p | -0.18 |
| 7534 | YWHAZ | hsa-mir-155 | -0.17 |
| 836 | CASP3 | hsa-let-7a | -0.17 |
| 5597 | MAPK6 | hsa-mir-26b | -0.17 |
| 4087 | SMAD2 | hsa-mir-155 | -0.15 |
| 9435 | CHST2 | hsa-mir-185 | -0.15 |
| 6678 | SPARC | hsa-mir-29a | -0.14 |
| 7316 | UBC | hsa-mir-20a | -0.13 |
| 1326 | MAP3K8 | hsa-mir-20a | -0.11 |
| 665 | BNIP3L | hsa-mir-23a | -0.1 |
| 948 | CD36 | hsa-mir-423-5p | -0.09 |
| 665 | BNIP3L | hsa-mir-23b | -0.08 |
| 6850 | SYK | hsa-mir-886-5p | -0.08 |
| 7534 | YWHAZ | hsa-let-7a | -0.08 |
| 3678 | ITGA5 | hsa-mir-30b | -0.08 |
| 4170 | MCL1 | hsa-mir-16 | -0.07 |
| 3586 | IL10 | hsa-mir-374a | -0.04 |
| 637 | BID | hsa-mir-26a | -0.04 |
| 3678 | ITGA5 | hsa-mir-27a | -0.03 |
| 1236 | CCR7 | hsa-let-7a | -0.02 |
| 2114 | ETS2 | hsa-mir-886-5p | -0.01 |

* Predicted target genes showing negatively correlated expression compared with miRNA expression data. Differentially expressed genes with at least 2-fold altered expression or temporal expression pattern (figure 1 B and C) and regarding expression data of each time point and MAH strain were considered. Target Scan and PITA were used as algorithms for target prediction using the web tool MAGIA.

** miRNA showing consistent expression over all time points and both MAH strains (10091/06 and 104) predicted to be regulators of differentially expressed genes.

*** Pearson correlation coefficient.
